# Reliable directional relaying for T-Connected Series-Compensated transmission lines

**DOI:** 10.1038/s41598-025-33564-9

**Published:** 2026-01-17

**Authors:** Mahmoud A. Elsadd, Ahmed R. Adly, Mahmoud M. Elgamasy

**Affiliations:** 1 Electrical Engineering Dept., Faculty of Engineering, Damanhour University, Damanhour, Egypt; 2 Nuclear Search Center, Egyptian Atomic Energy Authority, Cairo, Egypt; 3 Electrical Engineering Dept., Faculty of Engineering, Menoufia University, Shebin El kom, Egypt

**Keywords:** Fault direction, Series-compensation, MOV, Transmission lines, Current inversion, Voltage inversion, Electrical and electronic engineering, Energy grids and networks

## Abstract

This article introduces an extensive directional relaying algorithm designed for series-compensated transmission lines. The proposed algorithm relies on observing the locus of the calculated impedance of the change of the positive-sequence circuit. Both the voltage and current signals in positive sequence circuit are observed to acquire the impedance perceived by the relay. The fault direction is identified by the quadrant in which the locus of the obtained impedance settles. The proposed algorithm is distinguished by its high performance under varying prefault power factor and active power flow direction. It is tested for a range of fault types, varying fault resistance values, diverse fault locations, and different compensation ratios. All tests have been conducted while considering the nonlinear behaviour of the shunt metal oxide varistor (MOV) with the series-compensation capacitor. Furthermore, the proposed scheme is validated with a T-connected multi-terminal Egyptian transmission system. It is extensively tested and compared with other relevant schemes in the literature.

**Supplementary Information:**

The online version contains supplementary material available at 10.1038/s41598-025-33564-9.

## Introduction

### Motivation

Transmission lines (TLs) have a vital function in facilitating the transfer of electrical power across distinct locations. As the demand for power generation continues to rise, TLs may face limitations in their ability to efficiently transmit the required amount of power. To tackle this problem, series capacitors are utilized to compensate for the line reactance and improve the capacity of the transmission lines^1–4^. On the other hand, directional overcurrent relays, together with breaker failure relays, are employed as local backup protection in transmission systems due to their relatively lower cost and faster response compared to primary protection schemes such as distance and current-differential relays^5,6^. However, the series compensation introduces challenges for protection engineers, as traditional protection mechanisms may not be suitable for the altered configuration during fault conditions^1–4^. This challenges is due to the non-linear behavior of the metal oxide varistor (MOV), which can cause current inversion, and voltage inversion under certain faults, leading to potential misoperation of directional protection functions.

T-connected transmission systems are increasingly employed to interconnect regional power grids, particularly in cases where renewable energy resources are integrated into the network, as in the Egyptian T-connected configuration linking the Qena and Suez grids via a 220 kV, 516 km transmission line^7^, which was investigated by some of the same authors. The system incorporates two major wind farms: Zaafarana (545 MW) and Gabal El-Zayt (850 MW), with the latter showing strong potential due to favorable wind conditions. Future plans aim to expand the generation capacity in the Gabal El-Zayt region to 4000 MW, underscoring the growing demand for a robust and efficient transmission infrastructure. Despite the high generation potential, the current design capacity of the transmission corridor is limited to 1500 MW^7^, creating a significant gap between renewable energy production and transmission capability. To address this bottleneck and support long-distance power transfer, one economical solution is the implementation of series capacitor compensation stations. However, the integration of series compensation with T-connected topology introduces challenges for traditional protection schemes, making accurate directional relaying essential to ensure system security and operational reliability. This paper focuses on developing an accurate directional relaying scheme to be incorporated into the backup protection of T-connected transmission systems with series compensators considering wind farm integration.

### Literature survey

In the pursuit of a more dependable and secure power system, ensuring the protection of Series-Compensated Transmission Lines (SCTLs) emerges as a paramount goal. Nonetheless, researchers encounter numerous challenges in this endeavour. These challenges originate from the utilization of a metal oxide varistor (MOV) for the protection of the series capacitor (SC), which exhibits a non-linear behaviour^8–12^. Consequently, specific fault scenarios may lead to current inversion, or voltage inversion^13–16^. With the protection relay located at the SC, the voltage is inverted if the impedance up to the faulty point is inductive, and the impedance beyond the relay point is capacitive^14–16^. On other cases, the current is inverted if the equivalent impedance up to the faulty point is capacitive^14–16^. As a result, protective devices such as the directional function encounter issues of improper operation across a range of conditions. Therefore, the development of a reliable directional relaying solution for T-connected series-compensated transmission lines remains a significant hurdle for engineers working in protection and control.

In^1^ and^2^, a proposed approach relies on detecting faults in series-compensated lines by observing changes in the positive-sequence current phase angle and the magnitude alterations of the positive-sequence voltage during a fault event. However, the presented criteria in^1^ are altered if the prefault active power flow is reversed and more validation is needed with different prefault power factor and with the presence of nonlinear MOV. The approach in^2^ accounts for the reversed active power flow and successfully identifies the direction of faults. However, the method in^2^ assumes that the prefault active power flow direction is determined prior to the fault occurrence which is not reliable enough particularly with multiterminal transmission system. In^17^, fault detection and identification on the transmission line are accomplished by inputting fundamental current and voltage signals into an artificial neural network (ANN). However, this method relies heavily on extensive data samples and training for knowledge representation, making it less adept at handling uncertainties within the transmission system. In^18–20^, a scheme is presented that employs four identifiers depending on both positive and negative-sequence components. These identifiers are incorporated through a voting algorithm for determining the fault direction. In^21^, the paper discusses the directional relaying for diverse fault types, considering the presence of a static synchronous compensator integrated at various positions during single pole tripping. This scheme relies on analyzing the fault and prefault phase angles of the apparent power in the positive-sequence circuit. In^22^, a scheme is introduced that utilizes three classifiers based on the positive-sequence circuit, combined with a voting algorithm, to estimate the fault direction in a thyristor-controlled series-compensated transmission line. In^23^ and^24^, a directional scheme is proposed for series-compensated transmission lines depending on the first slope of the energy of the fault. This approach detects forward faults as having a negative initial change in the energy of the fault and reverse faults as having a positive initial change. In^25^, a fault classification scheme based on sub cycle power frequency and a method to identify the fault direction are presented for SCTLs. This method depends on the investigation of the initial alterations in voltage and current patterns caused by a fault. Nonetheless, it requires specifically tailored sensors and a high sampling rate. In^26^, the paper evaluates the performance of directional relaying applied to SCTLs, where the voltages are captured at the source side of the line beyond the included SC. Multiterminal transmission lines are considered in various schemes^27,28^. In^27^, a negative-sequence network-based method is introduced for accurate fault location in double-circuit multi-terminal transmission lines, where the need for communication links is eliminated and the number of required relay elements is reduced. In^28^, a non-pilot fault location element for series-compensated double-circuit lines is proposed, using a selected KVL loop in the negative-sequence network while considering shunt capacitances. However, the methods presented in^27,28^ focus on fault location determination. Various schemes have considered the impacts of series compensation and wind farm integration^29–31^. In^29^, the impacts of series compensation and wind farm integration on distance relays are investigated, and an intelligent relaying scheme based solely on current measurements is proposed, in which fault detection is performed using the signs of half-cycle magnitude differences of positive-sequence currents, and fault classification is carried out using Fourier–Bessel series expansion -based features and a bagging ensemble classifier. In^30^, a filter-assisted protection scheme is proposed for TCSC-compensated transmission lines connected to large-scale DFIG-based wind farms, where the dynamic impedance variation introduced by the TCSC and the complexities of wind integration are addressed. In^31^, a protection scheme is proposed for TCSC-compensated lines connected to DFIG-based wind farms, where fault detection uses the sign of half-cycle superimposed positive-sequence current, fault classification is performed via an EMD-assisted random forest, and fault location is estimated using a modified impedance method. In^32^, a directional protection method for transmission lines based on a positive impedance approach is presented, using instantaneous positive sequence voltage and current components to construct a Z matrix. Although the effectiveness of the method is evaluated across various power system configurations, it does not address T-connected series-compensated transmission lines. Therefore, the development of an accurate directional relaying scheme for integration into the backup protection of T-connected transmission systems with series compensation—considering the impact of wind farm integration—remains a key challenge for protection and control engineers and constitutes the primary motivation for this work.

### Contributions and paper organization

The primary contribution of this paper is the development of a directional relaying algorithm specifically designed for T-connected series-compensated transmission lines, with consideration of the impact of wind farm integration. By leveraging the observed equivalent impedance in the change of the positive-sequence circuit during fault conditions and employing a quadrant-based approach, the proposed algorithm effectively identifies fault direction, demonstrating its versatility across diverse fault types, different values of the fault resistance, varied fault distances, and compensation ratios. Notably, the proposed methodology accounts for the nonlinearity of the shunt metal oxide varistor in conjunction with the series-compensation capacitor, ensuring its reliability under realistic operating conditions. Furthermore, rigorous validation against a multiterminal transmission system and including scenarios of different prefault power factor, and reversed prefault active power flow showcases the algorithm’s superior performance when compared to existing schemes in the literature. This research represents a significant contribution to the field of power system protection, enhancing the security and reliability of modern electrical grids.

The remainder of the paper is organized as follows: Sect. “Proposed directional relaying algorithm” presents the proposed directional relaying algorithm. Sect. “Validation results” discusses the validation results. A comparison with other methods from the literature is provided in Sect. “Comparison with other methods in literature”. Sect. “Reliability evaluation” evaluates the reliability of the proposed approach. Finally, Sect. “Conclusion”concludes the paper.

## Proposed directional relaying algorithm

**Fig. 1:**
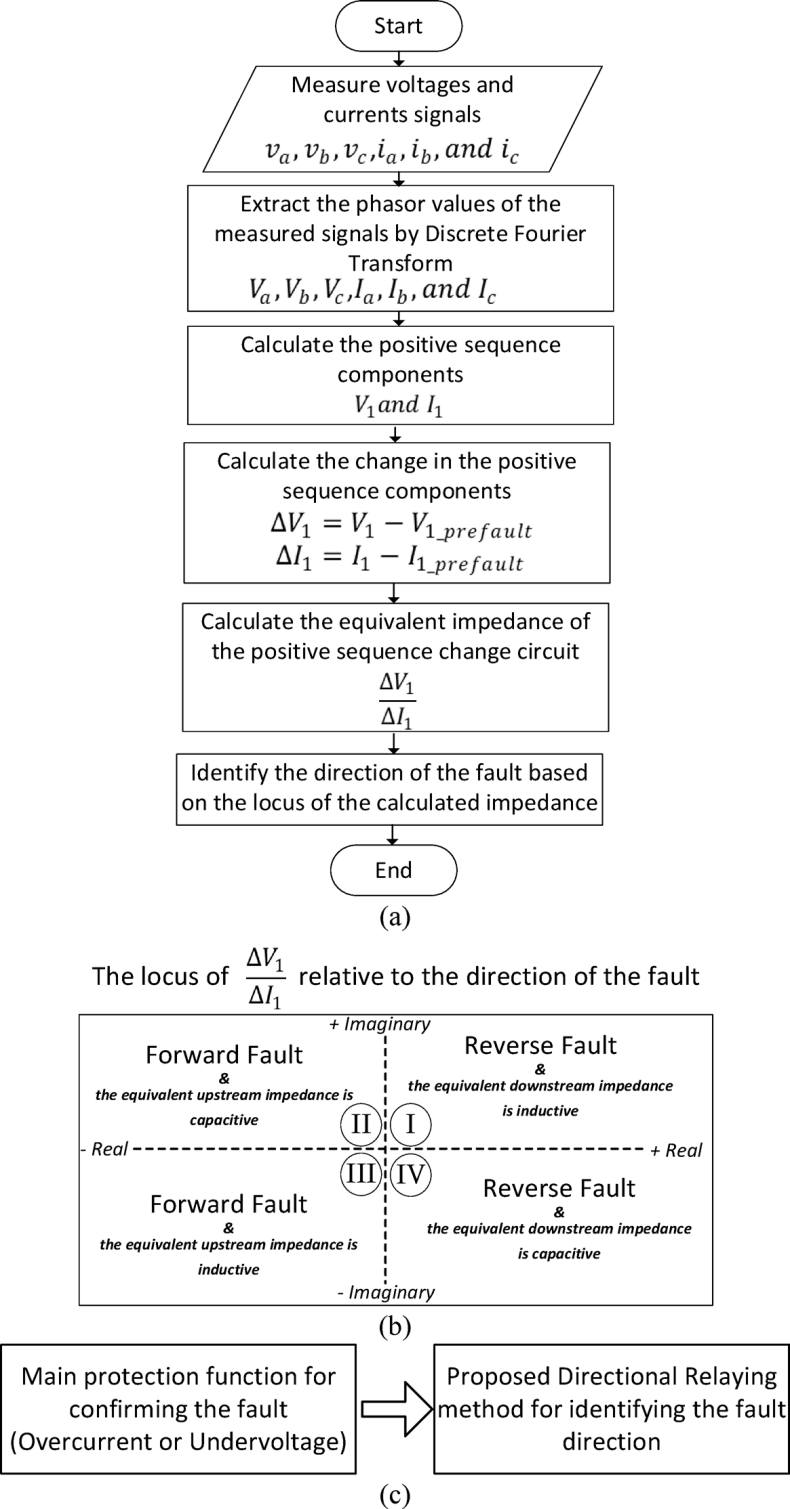
The proposed directional relaying algorithm; (**a**) The proposed algorithm; (**b**)The locus of $\Delta V_{1} /\Delta I_{1}$ under different fault directions; (**c**) coordination between the proposed directional relaying method and the main protection function (like overcurrent or undervoltage).

The proposed directional relaying algorithm relies on computing the equivalent impedance resulting from the alteration in the positive-sequence circuit. To determine the fault direction, the impedance locus obtained is analyzed. Figure 1a illustrates the proposed directional algorithm, where the changes in positive-sequence signals, ${\Delta V}_{1}$ and ${\Delta I}_{1}$, are computed for voltage and current, respectively. Subsequently, the equivalent impedance is determined through dividing ${\Delta V}_{1}$ by ${\Delta I}_{1}$. Figure 1b shows the possible loci of the calculated impedance, considering both forward and reverse fault conditions and accounting for inductive or capacitive nature of the impedance. If the locus falls within the second or third quadrants, it indicates a forward fault direction relative to the relay. Conversely, if the locus is situated in the first or fourth quadrants, it means a reverse fault direction.

It is important to note that the proposed directional relaying method is specifically designed for identifying the direction of the fault, rather than detecting the fault itself. In practical applications, relying solely on a single protection function may not ensure fast and reliable fault detection, particularly in systems with high renewable penetration and complex fault scenarios. Therefore, a combined protection scheme is often employed, where traditional protection functions—such as overcurrent or undervoltage—are used to detect and confirm the presence of a fault. Once the fault is detected, the proposed directional method is activated to determine its direction, which is essential for selective and accurate isolation. This coordinated approach enhances the overall protection performance and ensures robust operation under a wide range of system conditions. Figure 1c illustrates the potential integration of traditional protection functions with the proposed directional relaying method. An important advantage of combining the proposed directional relaying method with a traditional protection function—such as overcurrent or undervoltage as a backup protection—is that it eliminates the need for defining distance-related boundaries or zones in the ${\Delta V}_{1}$/${\Delta I}_{1}$ plane, as required in conventional distance protection schemes.

The proposed method is versatile and can be applied to all compensated transmission circuits under various fault types. The analysis of the change of the positive-sequence circuit of a two-terminal and T-connected compensated transmission systems is illustrated as follows:

### The change of the positive-sequence circuit of a two-terminal compensated transmission system under different fault types

#### Change of positive-sequence circuit under three-phase fault

**Fig. 2:**
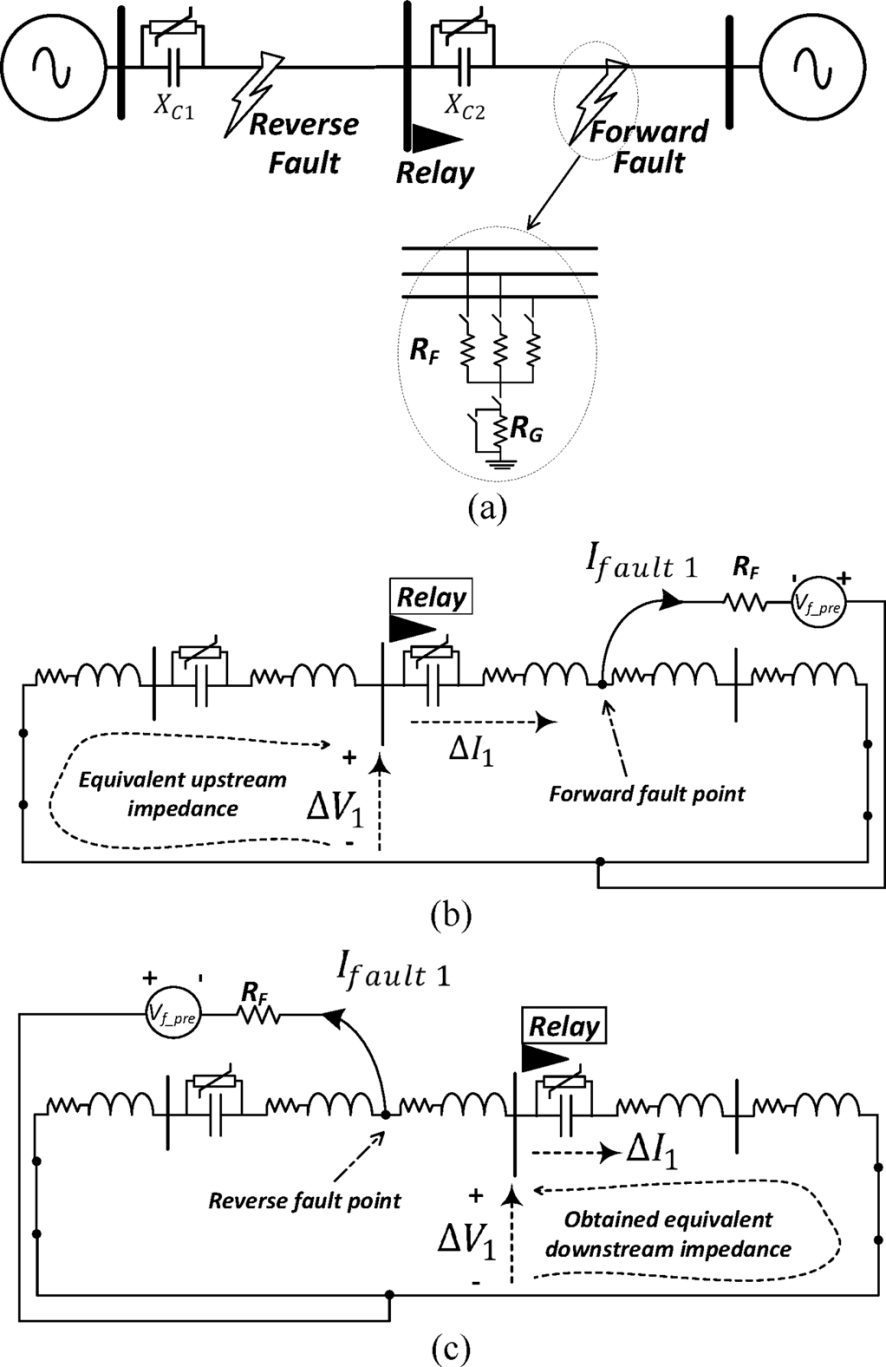
(**a**) Two-terminal transmission circuit configuration with the proposed relay located at the middle busbar; the connection of the change of positive-sequence circuit showing the equivalent impedance observed by the proposed relay: (**b**) under forward three-phase fault; (**c**) under reverse three-phase fault.

Figure 2a illustrates the diagram of a series-compensated transmission circuit where the proposed directional relay is positioned at the middle busbar. The change of the positive-sequence circuit under forward and reverse three-phase fault conditions is elaborated in Fig. 2b, c, respectively. The obtained equivalent impedance by the relay differs in both cases. In Fig. 2b, the equivalent impedance observed by the relay is located upstream of the relay, while in the other case, depicted in Fig. 2c, the equivalent impedance observed by the relay is located downstream of the relay. Under forward fault condition, the locus of $\Delta {V}_{1}/\Delta {I}_{1}$ settles in the third or second quadrant if the upstream impedance is inductive or capacitive, respectively. On the other hand, if the fault is in the reverse direction, the locus of $\Delta {V}_{1}/\Delta {I}_{1}$ settles in the first or fourth quadrant if the downstream impedance is inductive or capacitive, respectively.

#### Change of positive-sequence circuit under asymmetrical faults

**Fig. 3:**
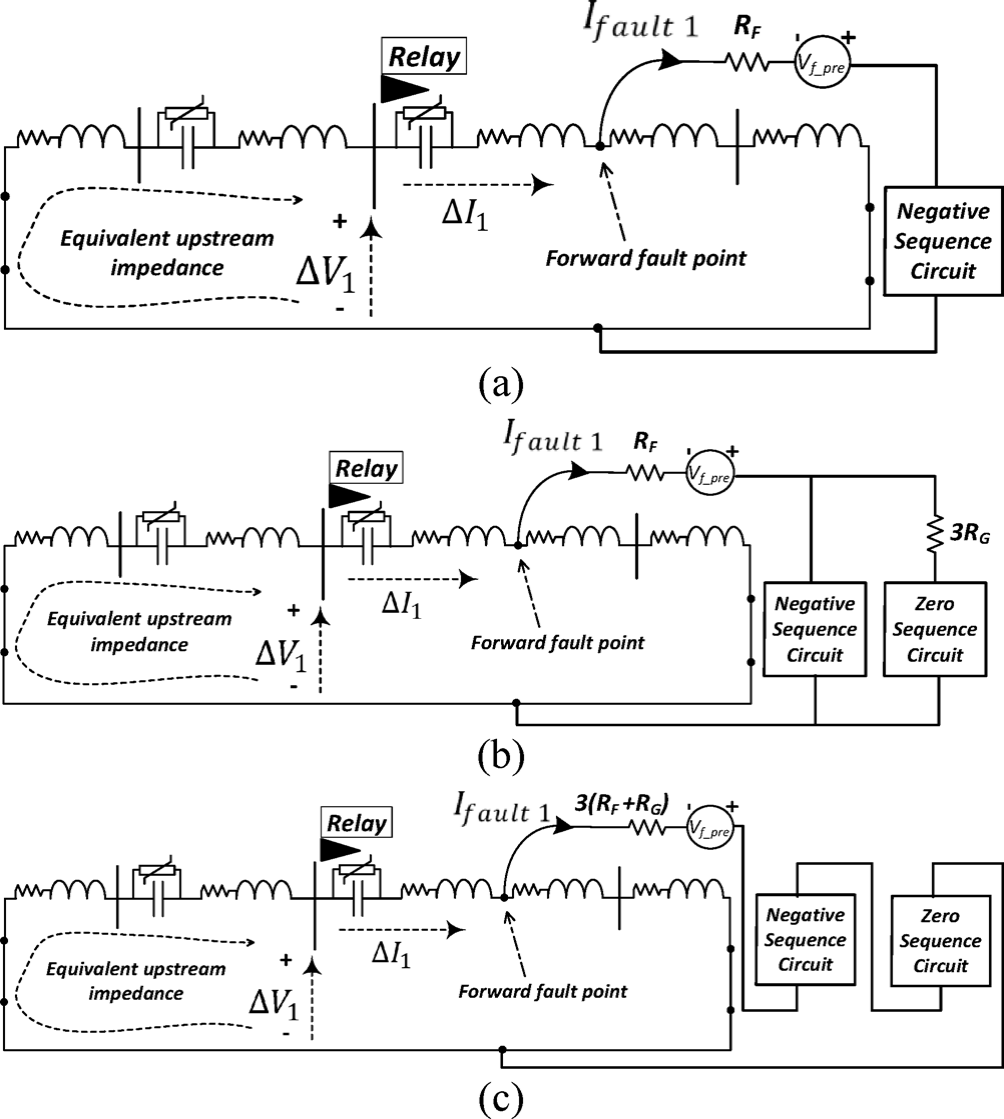
The connection of the change of positive-sequence circuit of a series-compensated transmission system under asymmetrical types of the fault including: (**a**) line-line fault; (**b**) line-line-ground fault; (**c**) line-ground fault.

Under asymmetrical fault conditions, the negative-sequence and zero-sequence circuits become active. For the sake of simplicity, only the forward fault condition is explained in this section under various asymmetrical fault types. Figures 3a–c show the connection between the positive-sequence circuit with other circuits under line-to-line, double line-to-ground, and line-to-ground fault cases, respectively. As depicted in Fig. 3, the equivalent impedance seen by the proposed relay during the forward fault is situated upstream of the relay. This means that the observed upstream impedance remains unaffected by the fault type which is a crucial advantage of the proposed method.

### The change of the positive-sequence circuit in a T-connected compensated transmission system with considering non-linear elements

**Fig. 4:**
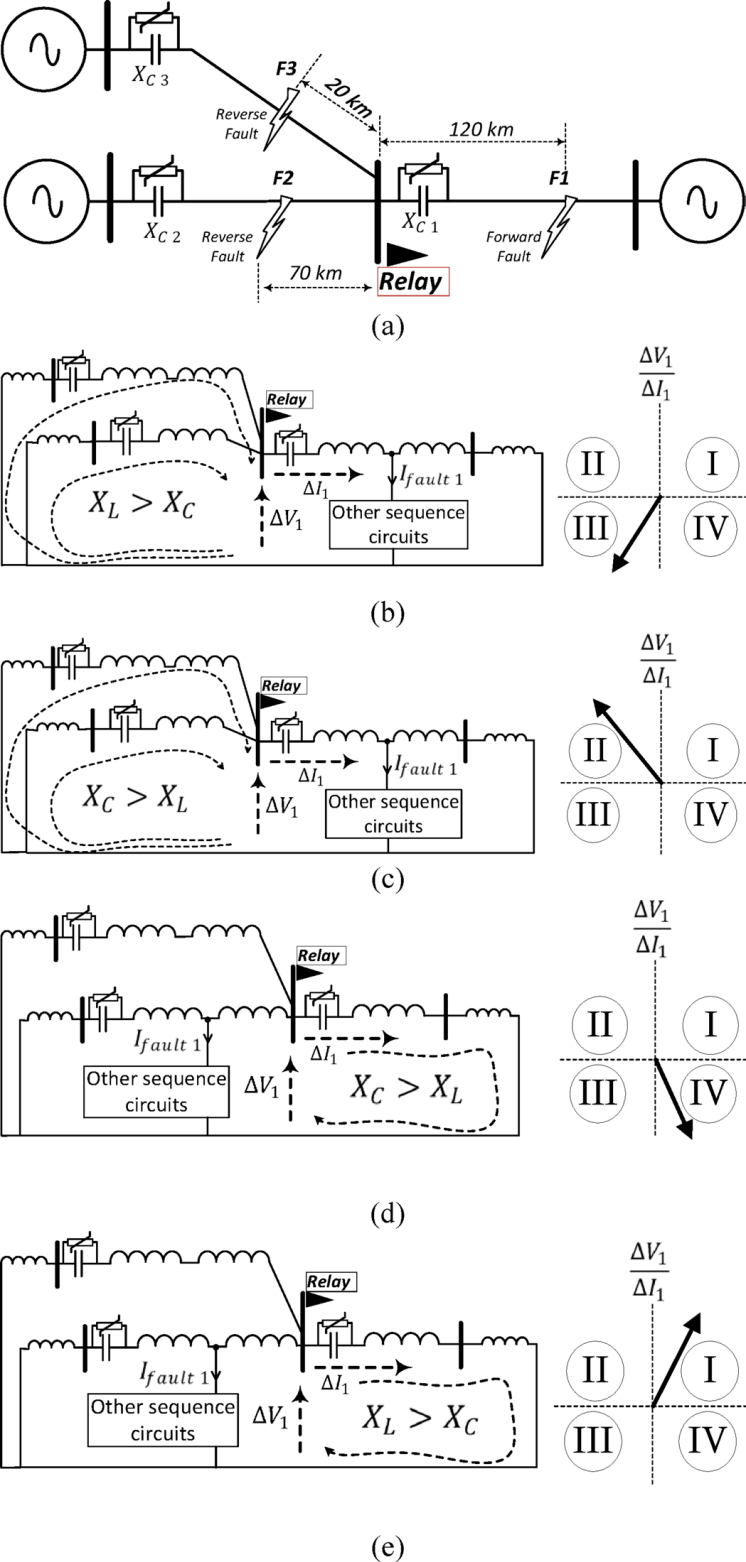
(**a**) T-connected transmission system configuration; locus of the observed impedance by the proposed relay under: (**b**) Forward fault condition with inductive equivalent impedance upstream of the relay; (**c**) Forward fault condition with capacitive equivalent upstream impedance; (**d**) Reverse fault condition with capacitive equivalent downstream impedance; (**e**) Reverse fault condition with inductive equivalent downstream impedance.

This subsection investigates all possible fault scenarios in T-connected compensated transmission system while considering the non-linearity of MOV. Figure 4a illustrates the considered T-connected system, with the proposed directional relay assumed to be located at the T point and providing protection for its downstream line. Each line is assumed to have a series capacitor with a parallel MOV for considering the effect of nonlinear behavior under fault conditions. All possible fault conditions are explained in the following.

#### Forward fault with inductive equivalent upstream impedance

Under forward fault condition, the impedance obtained by the relay reflects the equivalent impedance upstream of the relay in the change of the positive-sequence circuit. If the fault resistance is low, this results in high fault currents and consequently, a high voltage-drop across the capacitor, which causes the MOV to operate. The operation of MOV under high fault currents reduces the equivalent capacitance. In this condition, if the equivalent upstream impedance is inductive, the impedance observed by the relay is situated in the impedance plane’s third quadrant, as depicted in Fig. 4b.

#### Forward fault with capacitive equivalent upstream impedance

Under forward fault condition, if the equivalent impedance upstream of the relay is capacitive, the locus of the observed impedance occupies the impedance plane’s second quadrant, as illustrated in Fig. 4c.

#### Reverse fault with capacitive equivalent downstream impedance

Under reverse fault condition, the seen impedance by the proposed relay is that downstream of the relay in the change of the positive-sequence circuit as illustrated in Fig. 4d. When the predominant reactance in the downstream direction is capacitive, the obtained impedance is positioned in the impedance plane’s fourth quadrant, as depicted in Fig. 4d.

#### Reverse fault with inductive equivalent downstream impedance

If the fault is reverse, and the downstream reactance is predominantly inductive, the locus of the observed impedance by the proposed relay is situated in the impedance plane’s first quadrant, as shown in Fig. 4e.

### Capability of the proposed relaying method to handle different aspects

The proposed directional relaying algorithm is capable of accurately identifying the fault direction under various system conditions. The locus of the operating variable ($\Delta {V}_{1}/\Delta {I}_{1}$) in the proposed method settles in the third or second quadrants for forward faults, and in the first or fourth quadrants for reverse faults. This behavior remains consistent regardless of the prefault conditions, system configuration, fault type, fault resistance value, fault location, compensation ratio, non-linear MOV, or uncertainty of parameters.

#### Prefaut power flow

The reliance on the change of the positive-sequence circuit, in the proposed method, effectively isolates the impact of the prefault conditions. Under fault conditions, the system can be represented as a combination of separate circuits: the change in the positive-sequence circuit and the prefault circuit. The prefault circuit is not included in the figures to maintain clarity in presentation. The separation between these two circuits ensures that the influence of prefault conditions is eliminated, resulting in a distinctive and consistent fault signature that remains valid under all operating circumstances. The change of the positive-sequence circuit is free from any sources within the loop measured by the relay and is independent of the prefault power flow.

#### Different system configurations

The proposed method is also applicable to different system configurations, not limited to the two-terminal case. Figure 4 illustrates the performance of the proposed algorithm in a three-terminal system. As shown, when a forward fault occurs, the locus of the equivalent impedance seen by the proposed relay can only appear in the third or second quadrants. Conversely, for a reverse fault, the locus is confined to the first or fourth quadrants.

#### Different fault types

Figure 2b illustrates the equivalent impedance seen by the proposed relay for a three-phase forward fault in a two-terminal system. Figure 3a–c show the equivalent impedance seen by the relay for forward L–L, L–L–G, and L–G faults, respectively. As observed from the figures, the equivalent impedance seen by the proposed relay corresponds to the portion of the line upstream of the relay. The behavior of the proposed relaying method is not affected by the fault type. If there is a slight deviation in the equivalent impedance for different fault types, the locus of ($\Delta {V}_{1}/\Delta {I}_{1}$) still settles only in the third or second quadrants under forward fault conditions, and in the first or fourth quadrants under reverse faults, regardless of the fault type.

#### Fault resistance value

The analysis presented in Figs. 2, 3, and 4 demonstrates that the fault resistance does not contribute to the equivalent impedance seen by the proposed relay. As shown in Fig. 2b, for a forward fault, the fault resistance is excluded from the impedance path observed by the relay. Similarly, in Fig. 2c, under reverse fault conditions, the impedance seen by the relay also does not include the fault resistance. This behavior remains consistent for different fault types, as illustrated in Fig. 3, and under various system configurations, as shown in Fig. 4.

#### Diverse fault locations

 The fault location does not influence the performance of the proposed relaying algorithm. As illustrated in Figs. 2b, 3 and 4b, c for forward fault conditions, varying the fault distance does not affect the equivalent impedance seen by the relay. The relay measures the impedance upstream of its location, which remains independent of the downstream network in the fault direction and therefore unaffected by the fault position.

 Similarly, for reverse fault conditions, the impedance seen by the relay downstream remains unchanged with the fault location, as shown in Figs. 2c and 4d, e.

#### Different compensation ratios/nonlinear MOV/uncertainty of parameters

 The nonlinearity of the MOV primarily influences the equivalent impedance seen by the proposed relay. When the MOV operates, the equivalent impedance varies depending on the fault type and severity; however, the proposed scheme effectively adapts to all possible operating conditions. The proposed method does not depend on the exact value of the impedance but rather on whether it is inductive or capacitive. Each fault direction corresponds to two specific quadrants of the impedance locus observed by the proposed relay. If the equivalent impedance is inductive under a forward fault, the impedance locus settles in the third quadrant, as shown in Fig. 4b. Conversely, if the impedance is capacitive under a forward fault, the locus shifts to the second quadrant, as illustrated in Fig. 4c. For reverse faults, when the equivalent impedance is capacitive, the locus appears in the fourth quadrant, as shown in Fig. 4d; and when it is inductive, the locus moves to the first quadrant, as seen in Fig. 4e. Therefore, variations in the compensation ratio, MOV operation, or parameter uncertainties do not affect the performance of the proposed directional relaying algorithm.

## Validation results

### Two-terminal compensated transmission system

A two-terminal 400 kV transmission system is simulated using Matlab/Simulink, configured as shown in Fig. 2a. The simulated transmission circuit has a total length of 300 km, with the relay installed at the middle busbar, dividing the circuit into two equal sections, each spanning 150 km. The system is 70% compensated, with capacitors equally distributed between the two sections. To protect each capacitor from overvoltage, metal oxide varistors (MOV) are employed. The transmission lines are represented by a distributed-parameters model, and the detailed parameters of the transmission circuit, grid parameters, and MOV are provided in the Supplementary Information. The MOV is modelled as a controlled current source connected in parallel with the capacitor, as illustrated in Fig. S1. The detailed parameters of the simulated grid are listed in Table S1, while the parameters of the transmission system are provided in Table S2 in the Supplementary Information. The sampling rate of the measured signals is set to 10 kHz.

**Fig. 5:**
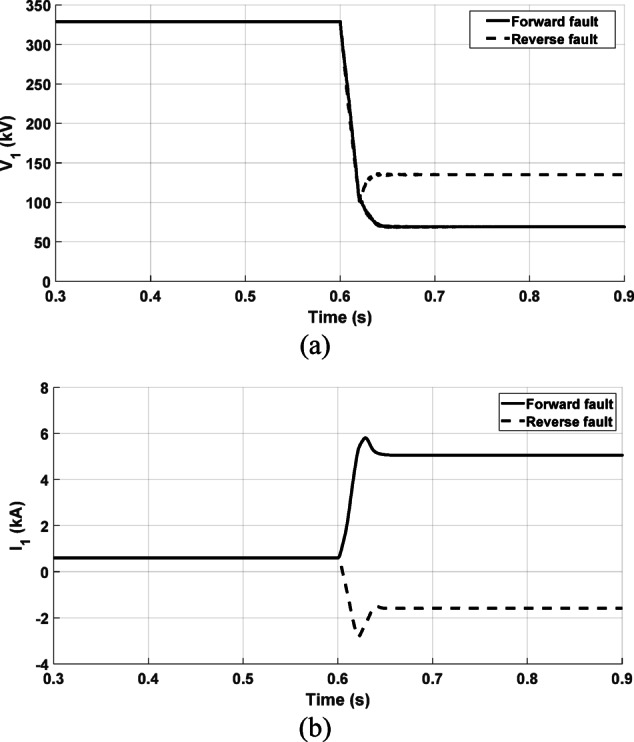
The measured signals at the relay point; (**a**) The positive sequence voltage measured at the relay point under forward and reverse faults at 70 km with referring to the relay point; (**b**) The positive sequence current measured at the relay point.

**Fig. 6:**
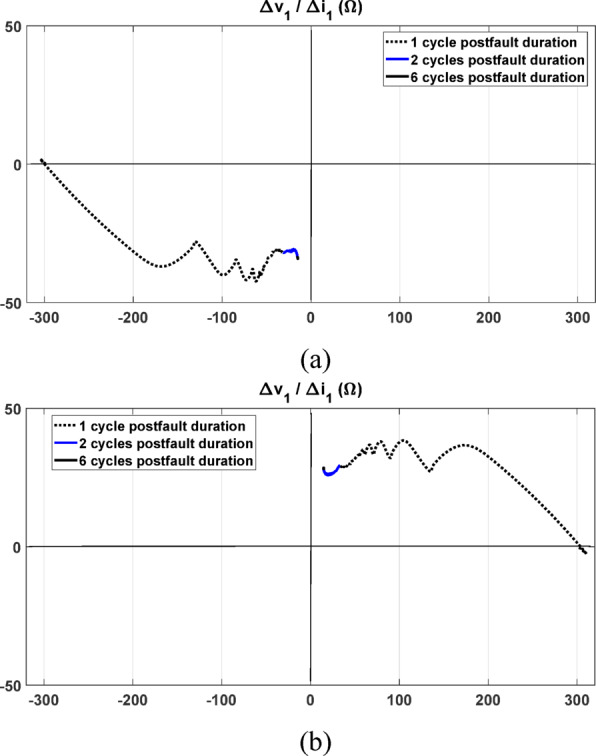
The obtained impedance by the proposed relay under forward and reverse fault conditions; (**a**) under forward fault condition at 70 km from the relay during the postfault 6 cycles; (**b**) under reverse fault condition at 70 km from the relay during the postfault 6 cycles.

The proposed directional relaying algorithm is subjected to testing under both forward and reverse fault cases. In both scenarios, the fault location is set at 70 km relative to the relay point. Figure 5 illustrates the measured positive-sequence components of the voltage and current seen by the proposed relay during the simulated three-phase fault. The fault direction is determined based on the locus of the equivalent impedance in the change of the positive-sequence circuit. Under the forward fault condition, Fig. 6a displays the locus of the impedance observed by the proposed relay. The locus is evidently situated in the impedance plane’s third quadrant, confirming the fault as forward. Settling in the third quadrant indicates that the upstream impedance is inductive. Conversely, Fig. 6b demonstrates the response of the proposed relay algorithm during a reverse fault condition. As shown, the locus of the seen impedance by the proposed relay settles within the first quadrant, which is a successful indication of the fault direction as a reverse fault.

It is worth noting that the locus of the obtained impedance is represented at various time durations. The dotted part of the line represents the impedance observed through relay measurements during the first cycle after fault occurrence, while the blue part of the line shows the response during the post-fault 2 cycles. The locus is recorded for a total duration of 6 cycles after the fault to indicate the possible maximum extent of the locus in the impedance diagram.

### Performance under different fault distances

**Fig. 7:**
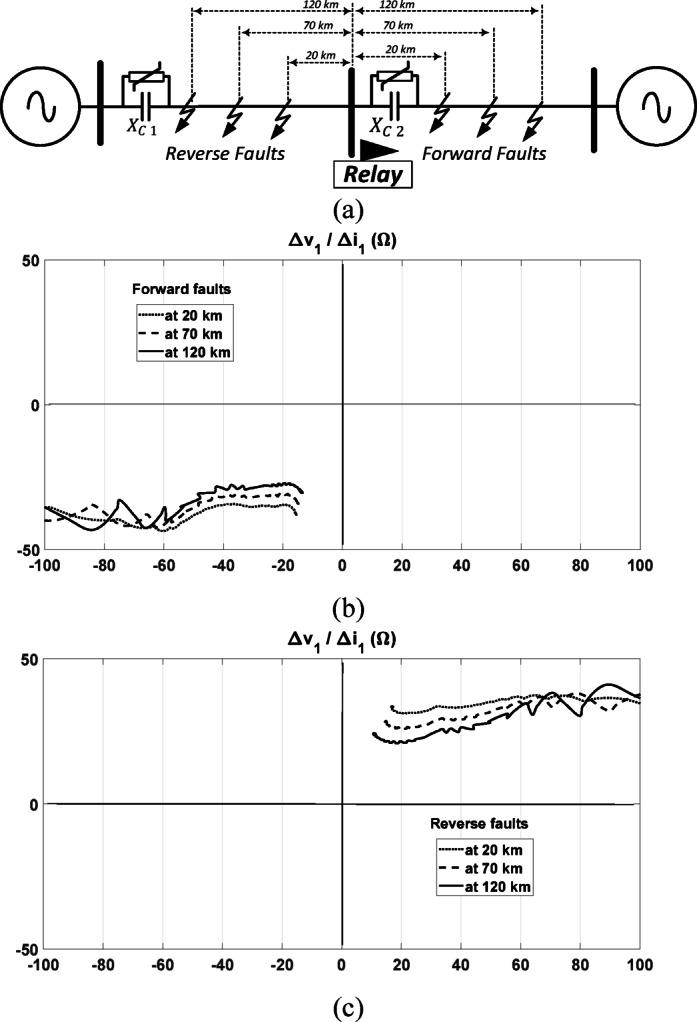
Response of the proposed method for (**a**) a series-compensated line under various fault locations; (**b**) impedance seen by the relay during forward faults; and (**c**) impedance seen during reverse faults.

It is worth highlighting that the impedance calculated through the proposed directional relay is scarcely influenced by the location of the fault particularly if there are not non-linear elements. This is since in the case of forward faults, the measured impedance by the relay represents the impedance upstream of the relay which is not altered by the location of the forward fault. Similarly, in the case of reverse faults, the obtained impedance by the relay represents the impedance downstream of the relay. This unique advantage of the proposed algorithm ensures that the fault location does not significantly impact the algorithm’s performance. With considering the non-linear behavior of MOV, the locus of the calculated impedance is changed depending on the degree of change of MOV impedance. To validate this point, it is assumed that each section in the transmission circuit has a non-linear MOV. Different simulations are conducted for three different fault locations at 20 km, 70 km, and 120 km relative to the relay point, for both forward and reverse fault conditions, as depicted in Fig. 7a.

Under various forward faults, the locus of the observed impedance by the relay consistently settles in the same quadrant, as seen in Fig. 7b. The three settling points are not the same due to the nonlinear behavior of the MOV, however, the decision of the proposed relay is not altered as the quadrant remains consistent under all three test cases.

Similarly, under reverse faults, the seen impedance settles in the same quadrant, regardless of whether the fault is close or distant from the relay, as shown in the results obtained in Fig. 7c. This demonstrates a significant advantage of the proposed directional relay, as it ensures reliable fault direction identification even with varying fault locations and with considering non-linear behavior of MOV.

### Performance considering different compensation ratios

**Fig. 8:**
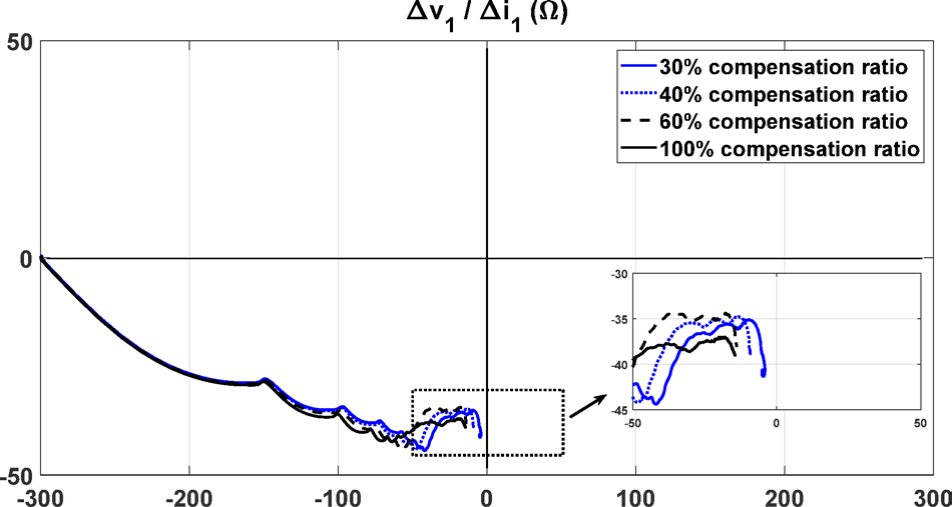
The seen impedance by the relay under forward fault condition with different compensation ratios.

The proposed algorithm is subjected to testing when the compensation ratio is varied. Forward three-phase fault cases are simulated at 20 km from the relay under different compensation ratios. As illustrated in Fig. 8, the algorithm’s response remains unaffected, with the locus eventually settling in the same quadrant. The slight variation in the final settlement point is attributed to changes in the capacitance ratio and the nonlinear behavior of the MOV. Despite these variations, the proposed algorithm consistently maintains its reliability in identifying fault direction.

### Performance under changed system parameters

Further testing of the proposed method was carried out considering variations in the transmission line parameters. The proposed method was tested with the line parameters increased by 50% and 75% under both forward and reverse fault conditions.

**Fig. 9:**
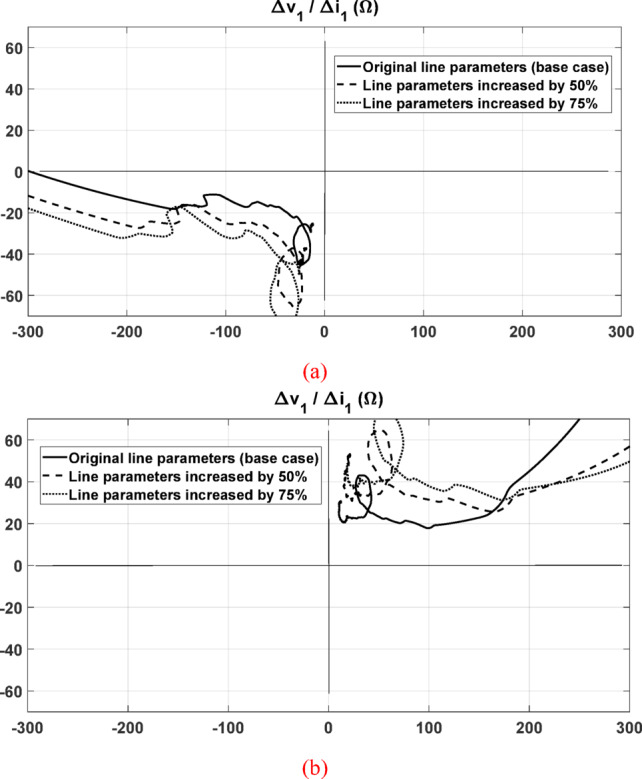
The seen impedance by the relay: (**a**) under forward L-G fault condition with changed system parameters; (**b**) under reverse L-L fault condition with changed system parameters.

L–G forward fault cases were simulated at a distance of 75 km from the relay while maintaining the compensation level at 70% of the original line parameters. The results, shown in Fig. 9a, confirm that the proposed relaying method remains effective, as the impedance locus settles in the third quadrant. It is clear that changing the system parameters results in a corresponding alteration of the impedance seen by the relay in the proposed method, approximately in the same ratio as the parameter change; however, this does not affect the decision of the proposed method, as the impedance locus remains in the same quadrant.

Other L–L reverse fault cases were simulated at 75 km from the relay point with the same compensation level. As presented in Fig. 9b, the proposed method continues to operate correctly, with the impedance locus settling in the first quadrant.

### Performance under different fault types and resistances in a T-connected transmission system

**Fig. 10:**
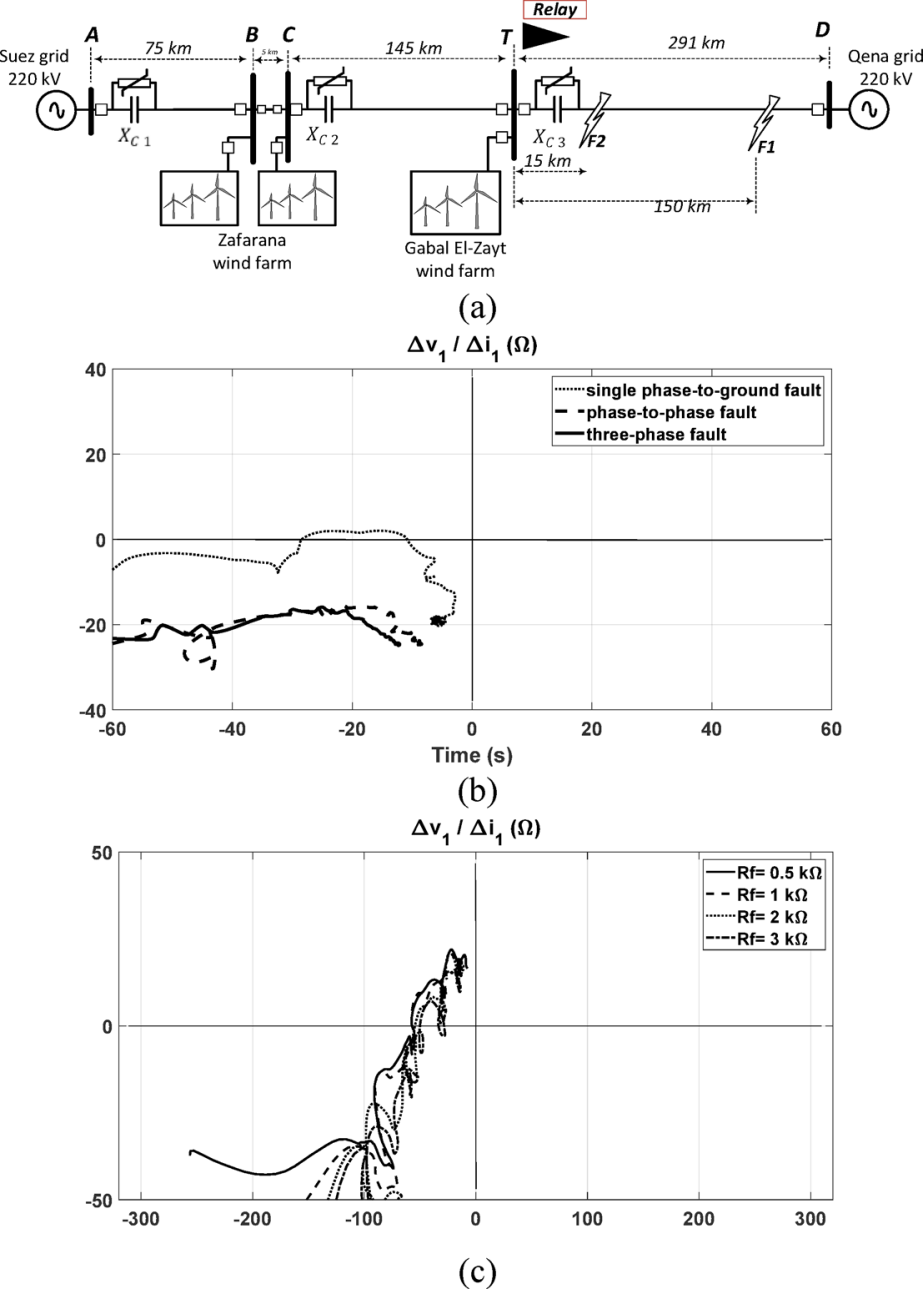
(**a**) Egyptian T-connected transmission system with integrated wind farm^7^; The response of the proposed method: (**b**) under different fault types at F1; (**c**) under L-G fault at F2 with high fault resistances.

The proposed directional relaying algorithm is evaluated in a T-connected system with the configuration shown in Fig. 10a. It is an Egyptian system for power transmission between two grids with having integrated wind farms. A shown in the figure it is 220 kV system connecting Qena and Suez grids via 516 km transmission circuit. There are two wind farms, the first one is Zaafarana wind farm with a capacity of 545 MW and located 75 km from the Suez side. The second wind farm has 850 MW capacity and located in Gabal El-Zayt region that is 291 km from Qena grid. This region is featured by its promising wind speed profile, and it is planned to increase the generating capacity of the wind farms to reach 4000 MW particularly in Gabal El-Zayt region. However, the transmission system is designed with a full capacity of 1500 MW^7^. In this study, we recommend raising the transmission system capability by employing series capacitor compensation stations. For considering different possible scenarios of load flow, each section is equipped with a series capacitor compensation station and the compensation ratios are selected such that the total series compensation ratio is 75%:

1 $$X_{C1} = 75\% X_{LAB} ,X_{C2} = 75\% X_{LCT} ,X_{C3} = 75\% X_{LTD} ,$$

The non-linear behavior of the circuit is considered where each capacitor is protected against overvoltage by using parallel MOV. The system in Fig. 10a is simulated by Matlab/Simulink where the wind farms are doubly fed Induction Generator (DFIG) type. The converters in the DFIG are simulated by its detailed model. Further details on the structure and control scheme of the DFIG-based wind power plant, including the overall configuration, converter control blocks, and PI controller gains for both the RSC and GSC, are provided in the Supplementary Information (Fig. S2 and Table S3). The sampling rate of the measured signals is set to 10 kHz.

The proposed directional relaying algorithm is tested under different fault types. With reference to the system in Fig. 10a, forward fault cases with different types are simulated including phase-to-ground, phase-to-phase, and Three phase faults. The proposed directional relay is tested by being installed at T point and providing protection for the line TD toward Qena grid. Figure 10b displays the obtained responses under different fault types, at *F1*, at 150 km away from the relay with solid fault conditions. As depicted, the obtained responses indicate that the fault is forward as the calculated impedance settles in the third quadrant. This means that the upstream equivalent impedance is inductive. The changes in the response are due to the asymmetrical operation of non-linear MOV with these fault conditions. The asymmetric operation of MOV under asymmetrical fault types affects the equivalent positive-sequence impedance of the MOV. One of the output points, in Fig. 10b, initially falls within the second quadrant, while the final value shifts to another quadrant. This does not present an issue, as both quadrants correspond to a forward fault, according to the quadrant interpretation shown in Fig. 1b. This behavior highlights a key advantage of the proposed method—its robustness against variations in the equivalent impedance resulting from the nonlinear behavior of the Metal Oxide Varistor (MOV). Despite the shift between quadrants, the method consistently identifies the fault as forward, confirming its reliability and practical applicability. In general, the proposed scheme is not negatively affected by the fault type, and the direction of the fault cases are all successfully identified as forward.

Furthermore, the response of the proposed scheme is tested under varying fault resistance values. A key advantage of the proposed scheme is its immunity to changes in the fault resistance value. This is supported by the analysis provided in Fig. 3, where the fault resistances (*RF* or *RG*) are connected outside the loop of the impedance obtained by the relay. Figure 10c shows the obtained results with the fault resistance value changed up to 3 kΩ under tested L-G fault conditions at F2. From the figure, the locus of the calculated impedance settles in the second quadrant after transitioning from the third quadrant. These responses result from the different equivalent impedances of the nonlinear MOV under these fault conditions. The appearance of both quadrants confirms that the fault is in the forward direction, demonstrating that the proposed method accurately identifies the fault direction while maintaining high reliability.

### Impact of renewable integration under varying compensation ratios

**Fig. 11:**
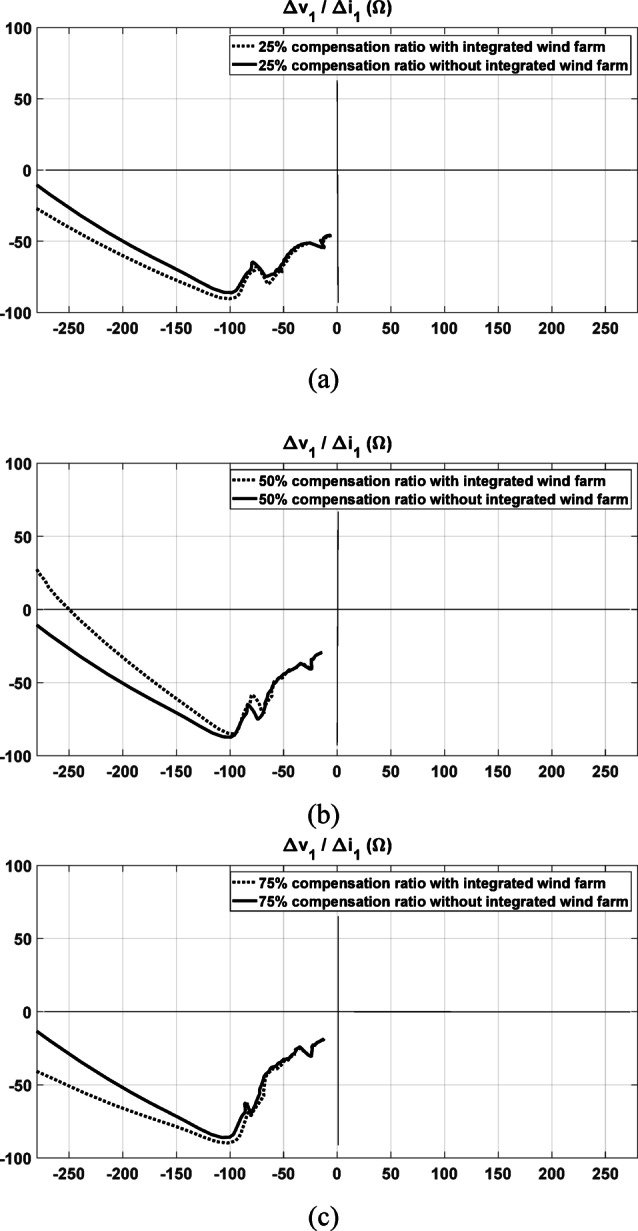
The response of the proposed method under forward three-phase fault at F1 both with and without the integration of the wind farms: (**a**) with 25% compensation ratio; (**b**) with 50% compensation ratio; (**c**) with 75% compensation ratio.

To investigate the effect of the renewable source under different compensation ratios, several test cases are conducted. The proposed directional relaying method is evaluated at compensation levels of 25%, 50%, and 75%. Each case is examined both with and without the integration of a wind farm. Figure 11a illustrates the results of the proposed directional relaying method when the system is 25% compensated, both with and without wind farm integration. This fault case is a forward three-phase fault at location F1 with a fault resistance of 150 ohms. As shown, only a slight deviation appears between the two responses, and the fault direction is accurately identified in both cases. This demonstrates the robustness of the proposed method in systems that include renewable sources. Similarly, Fig. 11b, c present the results for 50% and 75% compensation levels, respectively. In all scenarios, the method consistently identifies the fault direction correctly, further confirming its reliability across varying compensation levels and operating conditions.

**Fig. 12:**
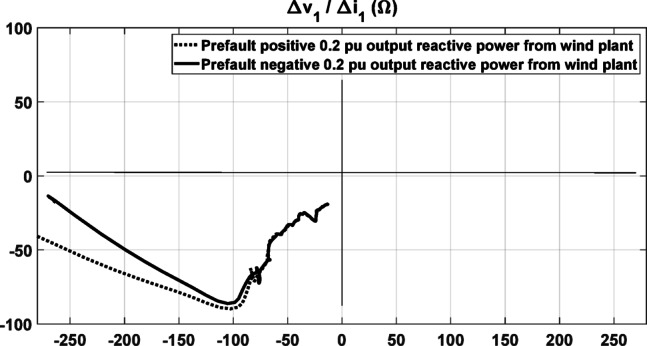
The response of the proposed method under forward three-phase fault at F1 with different controlled prefault output reactive power from the wind plant.

Furthermore, the proposed directional relaying method is tested under different controlled levels of prefault reactive power output from the DFIG. This test case considers a forward three-phase fault at location F1 with a fault resistance of 150 Ohms. As illustrated in Fig. 12, the fault direction is accurately identified in both scenarios. Therefore, the variation in prefault reactive power does not adversely impact the performance of the proposed relaying method, confirming its robustness under different operating conditions.

## Comparison with other methods in literature

The schemes presented in^1^ and^2^ rely on two criteria for determining the direction of the fault. The first criterion is the change in the positive-sequence current angle ($\Delta \varnothing$), and the second one is the change in the magnitude of the positive-sequence voltage ($\Delta \left|{V}_{1}\right|)$. The summarized criteria from^1^ are presented in Table 1. Nevertheless, these criteria are not universally applicable, as their effectiveness is influenced by the prefault power factor and MOV operation. A comprehensive comparison study is conducted to evaluate the performance under different conditions.

**Table 1:** The adopted criteria in^1^ and^2^ for identifying the fault direction.

$\Delta \emptyset$	$\Delta \left| {V_{1} } \right|$	Fault direction
Positive	Negative	Reverse
Positive	Positive	Forward
Negative	‘Do not care’	Forward

### Evaluating criteria presented in^1^ and^2^

**Fig. 13:**
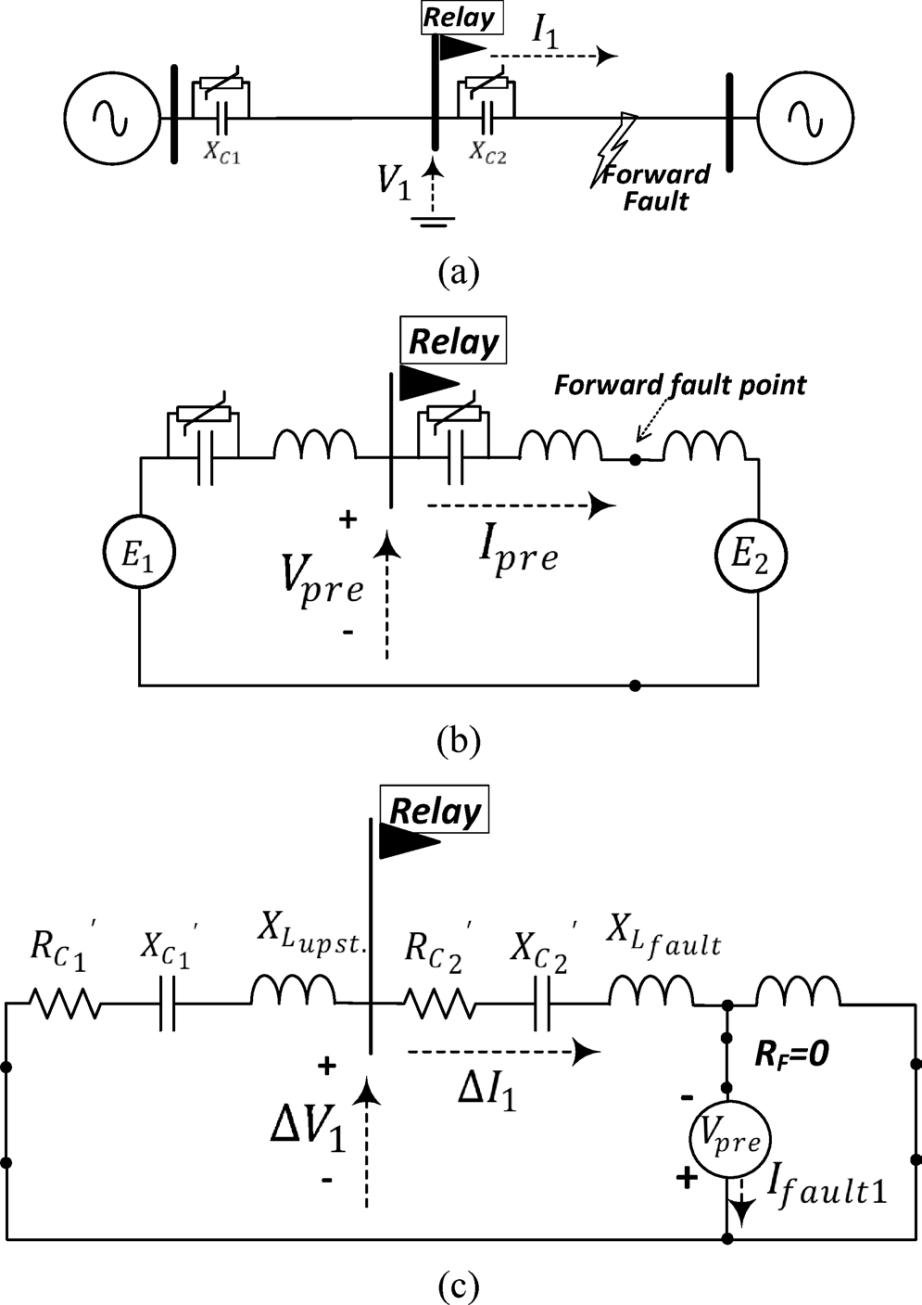
(**a**) A test system for evaluating the criteria in^1^ and^2^; (**b**) the prefault equivalent circuit; (**c**) the change of the positive-sequence circuit under forward solid three-phase fault condition.

**Fig. 14:**
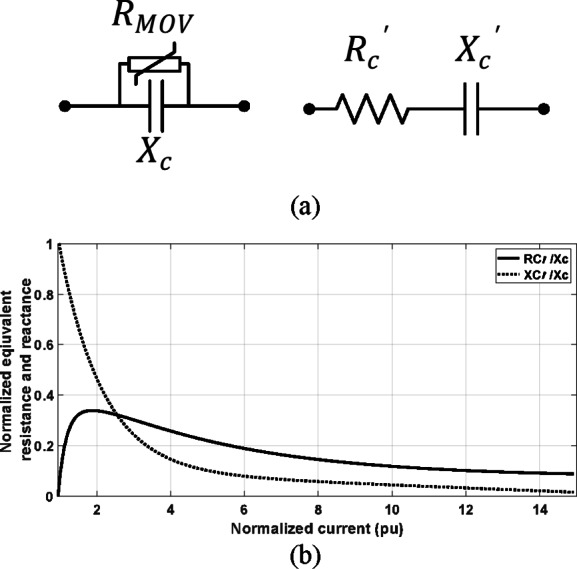
(**a**) The linearized model of the compensation capacitor and MOV; (**b**) The values of the linearized resistance and capacitance as a function of the current^33^.

The criteria presented in^1^ and^2^ are analytically evaluated by considering the system in Fig. 13a. The prefault equivalent circuit of the system is presented in Fig. 13b indicating the assumed forward fault point. The inductive reactance is for representing the impedance of the transmission system and sources. For simplicity, the fault is assumed solid three-phase type. Figure 13c shows the change of the positive-sequence circuit considering this fault type. The series capacitor and the parallel MOV are represented by its linearized equivalent impedance^33^ as in Fig. 14.

In Fig. 13c, ${V}_{pre}$ is the prefault voltage of the fault point, ${X}_{{L}_{upst.}}$ is the equivalent inductance of the transmission system and source upstream the relay, ${X}_{{L}_{fault}}$ is the inductive reactance of the transmission system up to the fault point with reference to the relay. ${{R}_{C1}}{\prime},{{X}_{C1}}{\prime},{{R}_{C2}}{\prime},{{X}_{C2}}{\prime}$ are the equivalent linearized series resistance and capacitive reactance of the compensator capacitor and MOV upstream and downstream the relay, respectively^33^.

The change of the positive-sequence current seen by the relay is denoted by $\Delta {I}_{1}$ and the change of the positive-sequence voltage at the relay point is represented by $\Delta {V}_{1}$. The change of the positive-sequence current is expressed as follows:

2 $$\Delta I_{1} = \frac{{V_{pre} }}{{R_{{C_{1} }}{\prime} - jX_{{C_{1} }}{\prime} + jX_{{L_{upst.} }} + R_{{C_{2} }}{\prime} - jX_{{C_{2} }}{\prime} + jX_{{L_{fault} }} }}$$

As depicted (3), (4) and (5), $\Delta I_{1}$ is represented as a phasor where its value, $\left| {\Delta I_{1} } \right|,$ and angle, $\theta_{1} ,$ are dependent on the fault location and MOV condition. The linearized resistance and capacitive reactance are highly affected by the fault current level.

3 $$\Delta I_{1} = \left| {\Delta I_{1} } \right|\angle \theta_{1}$$

4 $$\Delta I_{1} = \frac{{\left| {V_{pre} } \right|}}{{\sqrt {\left( {R_{{C_{1} }}{\prime} + R_{{C_{2} }}{\prime} } \right)^{2} + \left( {X_{{L_{upst.} }} + X_{{L_{fault} }} - X_{{C_{1} }}{\prime} - X_{{C_{2} }}{\prime} } \right)^{2} } }}$$

5 $$\theta_{1} = - \tan^{ - 1} \frac{{X_{{L_{upst.} }} + X_{{L_{fault} }} - X_{{C_{1} }}{\prime} - X_{{C_{2} }}{\prime} }}{{R_{{C_{1} }}{\prime} + R_{{C_{2} }}{\prime} }}$$

To evaluate the criteria in^1^ and^2^, the positive-sequence current seen by the relay needs to be analysed. $I_{1}$ is the positive-sequence current seen by the relay under fault condition. It is obtained by adding the prefault current with the change of the positive-sequence current at the relay point as follows:

6 $$\left| {I_{1} } \right|\angle \emptyset_{2} = \left| {I_{pre} } \right|\angle \emptyset_{1} + \Delta I_{1} \angle \theta_{1}$$

7 $$\Delta \emptyset = \emptyset_{2} - \emptyset_{1}$$

8 $$\Delta \emptyset is proportional to (\theta_{1} - \emptyset_{1} )$$

$\emptyset_{2}$ is the angle of the positive-sequence current seen by the relay after the fault occurrence and $\emptyset_{1}$ is the prefault one. The criteria in^1^ and^2^ depends on the change of this angle $\Delta \emptyset .$ From the above equations, the change in the angle of the positive-sequence current, $\Delta \emptyset ,$ is dependent on how the angles $\theta_{1}$ and $\emptyset_{1}$ are relatively allocated. If $\theta_{1}$ is higher than $\emptyset_{1}$*,* the change in the angle of the positive-sequence current, $\Delta \emptyset ,$ would be positive. $\emptyset_{1}$ is determined by the prefault power factor value and $\theta_{1}$ is dependent on the fault location and the status of MOV under fault condition. This means that the prefault power factor is a dominant factor in identifying the change of $\Delta \emptyset$ which is the first criterion in^1^ and^2^. Both studies have not investigated this point.

To evaluate the second criterion in^1^ and^2^, the positive-sequence voltage should be analysed. With reference to the equivalent circuit in Fig. 13c, the change of the positive-sequence voltage at the relay point is calculated as follows:

9 $$\Delta V_{1} = - 1*\Delta I_{1} *\left( {R_{{C_{1} }}{\prime} - jX_{{C_{1} }}{\prime} + jX_{{L_{upst.} }} } \right)$$

10 $$\Delta V_{1} = \frac{{ - 1*V_{pre} *\left( {R_{{C_{1} }}{\prime} - jX_{{C_{1} }}{\prime} + jX_{{L_{upst.} }} } \right)}}{{R_{{C_{1} }}{\prime} - jX_{{C_{1} }}{\prime} + jX_{{L_{upst.} }} + R_{{C_{2} }}{\prime} - jX_{{C_{2} }}{\prime} + jX_{{L_{fault} }} }}$$

11 $$\Delta V_{1} = \left| {\Delta V_{1} } \right|\angle \delta_{1}$$

12 $$\delta_{1} = \pi + \theta_{1} + \tan^{ - 1} \frac{{X_{{L_{upst.} }} - X_{{C_{1} }}{\prime} }}{{R_{{C_{1} }}{\prime} }}$$

The value, $\left| {\Delta V_{1} } \right|,$ and angle, $\delta_{1} ,$ are dependent on the fault location and MOV condition as well. The positive-sequence voltage at the relay point is calculated by adding the prefault change phasors $V_{pre} {\mathrm{and}} \Delta V_{1}$.

13 $$V_{1} = V_{pre} \angle 0 + \left| {\Delta V_{1} } \right|\angle \delta_{1}$$

14 $$\Delta \left| {V_{1} } \right| = \left| {V_{1} } \right| - \left| {V_{pre} } \right|$$

With assuming the prefault voltage at the relay as a reference, the change in the magnitude of the voltage $\Delta \left| {V_{1} } \right|$ is determined based on the angle $\delta_{1} .$ If $\left| {\delta_{1} } \right|$ is increased more than $\frac{\pi }{2}$, the value of the positive-sequence voltage would be reduced, and if it is lower than $\frac{\pi }{2}$, the value of the positive-sequence voltage would be increased. The angle is dependent on the upstream impedance including the upstream line $X_{{L_{upst.} }}$ and $R_{{C_{1} }}{\prime}$, $X_{{C_{1} }}{\prime}$ which are determined by the upstream MOV status. Both studies in^1^ and^2^ have not extensively investigate the effect of MOV operation along with the prefault power factor.

### Comparison study considering different prefault power factor

#### Response under forward three-phase fault with prefault leading power factor

**Fig. 15:**
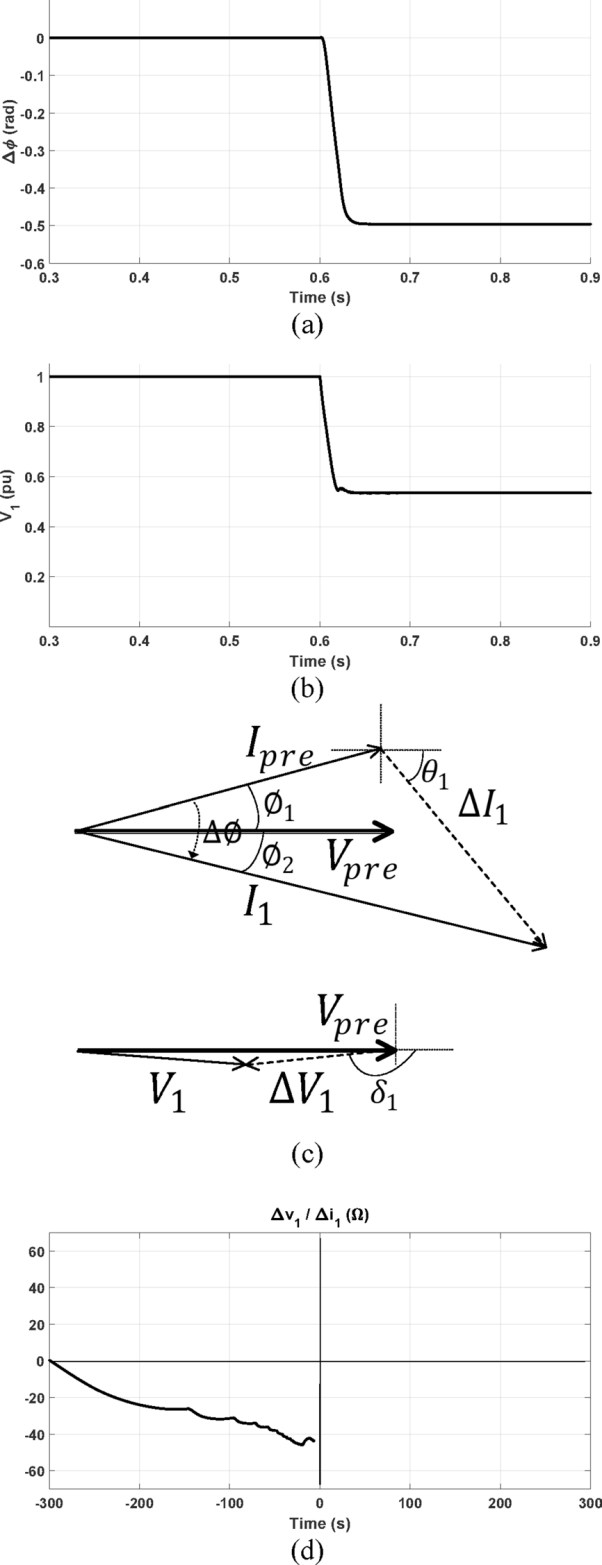
Obtained results under forward three-phase fault with prefault leading power factor (**a**) $\Delta \emptyset$ (**b**)The magnitude of $V_{1}$; (**c**) prefault and postfault phasors of current and voltage at the relay point; (**d**) proposed method result.

The directional relaying algorithms are tested with the system compensated by 50%. The two-terminal system in Fig. 13a is considered for this study. A prefault active and reactive power at the relay location are 2689 MW and − 718.5 MVAR with the power factor is 0.96 leading. A forward solid three-phase fault, with fault resistance of 0.1 Ω, is simulated at 15 km distance from the relay point. As depicted in Fig. 15, the methods proposed in^1^ and^2^ successfully identify the fault direction as a forward fault. This success is attributed to the negative change in both the positive-sequence current’s phase angle and the positive-sequence voltage’s magnitude as shown in Fig. 15a, b, respectively. Figure 15c shows the prefault and postfault phasors of current and voltage at the relay point under this fault condition. As shown, the significant negative value of the angle $\theta_{1}$ results in a negative change of $\Delta \emptyset ,$ and the magnitude of the positive-sequence voltage, $V_{1} ,$ is reduced as $\left| {\delta_{1} } \right|$ is more than $\frac{\pi }{2}$.

The proposed method excels in determining the fault direction when the locus of the calculated impedance settles in the third quadrant, indicating a forward fault as shown in Fig. 15d.

#### Response under forward three-phase fault with prefault lagging power factor

**Fig. 16:**
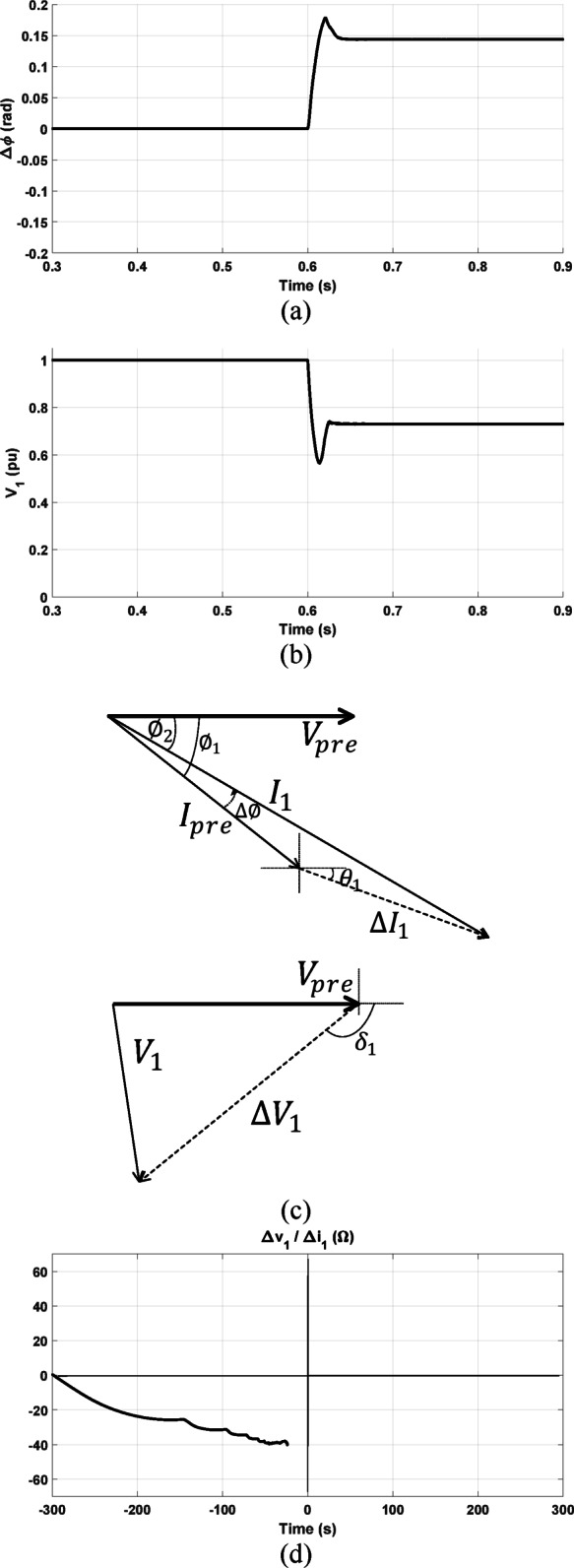
Obtained results under forward three-phase fault with prefault lagging power factor (**a**) $\Delta \emptyset ,$ (**b**)The magnitude of $V_{1}$ (**c**) prefault and postfault phasors of current and voltage at the relay point; (**d**) proposed method result.

The directional algorithms are tested considering the same forward fault condition but with changing the prefault power factor to be 0.78 lagging. As shown in Fig. 16a, b, the criteria presented in^1^ and^2^ gives an incorrect decision. As shown, the change of the positive-sequence current’s phase angle is positive and the positive-sequence voltage’s magnitude is reduced. The phasors of measured current and voltage signals at the relay point are indicated for this fault condition in Fig. 16c. As shown, in this case $\Delta {I}_{1}$ is leading $I_{pre}$, $\emptyset_{1}$ is more negative than $\theta_{1} .$ This results in making $I_{1}$ leads $I_{pre}$ and a positive value of $\Delta \emptyset$ is obtained. Also, the magnitude of the positive-sequence voltage, $V_{1} ,$ is reduced as $\left| {\delta_{1} } \right|$ is more than $\frac{\pi }{2}$. According to^1^ and^2^, this fault condition is diagnosed as a reverse fault condition which is an incorrect decision. However, the proposed method in this paper is not affected by the prefault power factor and the fault direction is correctly identified as forward where the locus of the calculated impedance settles in the third quadrant as seen in Fig. 16d.

#### Response under other forward fault conditions

**Fig. 17:**
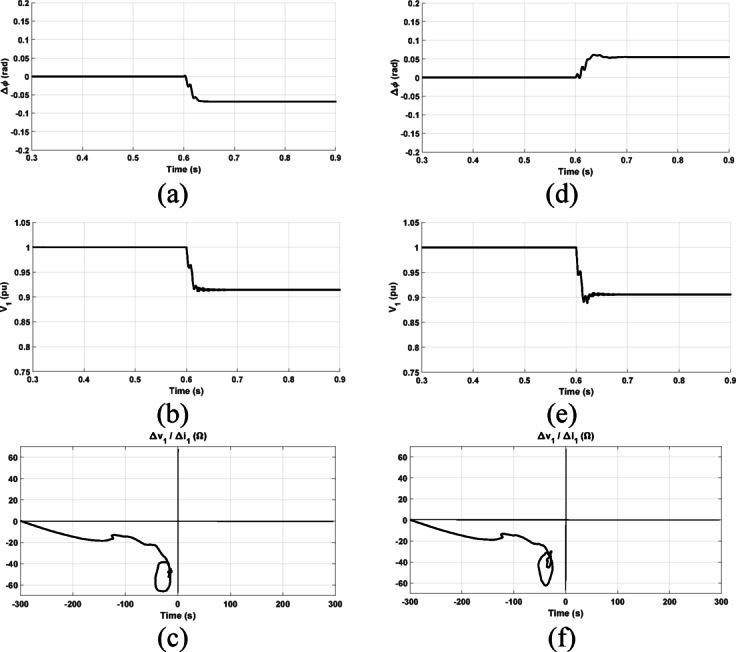
Obtained results under forward phase-to-ground fault conditions: (**a**–**c**) are with prefault leading power factor of 0.96; and (**d**–**f**) are with prefault lagging power factor of 0.94.

The directional relaying algorithms are tested with other fault cases with different conditions. A forward line-to-ground fault with fault resistance of 10 Ω is simulated at 15 km distance from the relay point. As depicted in Fig. 17, the methods proposed in^1^ and^2^ successfully identify the fault direction as a forward fault if the prefault power factor is leading. However, they fail in identifying the fault direction if the prefault power factor is lagging. Figure 17a, d indicate how the change in the angle of the positive-sequence current is altered with changed prefault power factor. Consequently, the methods in^1^ and^2^ are not reliable enough.

Figures 17b, e show the profile of positive-sequence voltage magnitude under the two considered prefault power factors. Furthermore, the loci of the calculated impedances using the presented approach under these considered power factors are illustrated in Fig. 17c, f. However, the response of the proposed method in this paper is not altered as the locus of each calculated impedance still settles in the third quadrant as seen in Fig. 17c, f regardless of the prefault power factor condition.

**Fig. 18:**
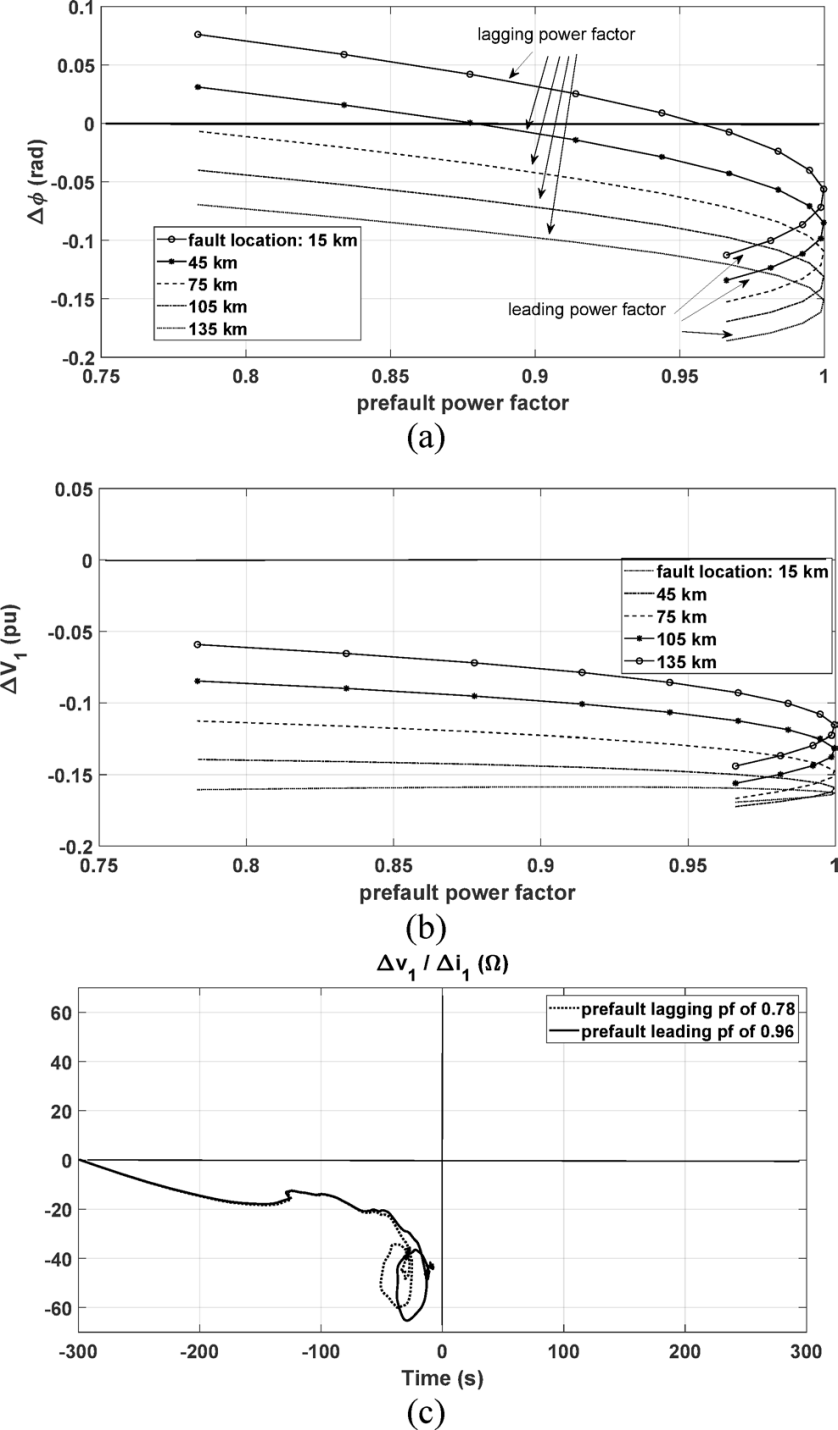
Comparison results under different prefault power factor values; (**a**) The change of the positive-sequence current’s phase angle $\Delta \emptyset$ with different fault distances; (**b**) The change of the positive-sequence voltage’s magnitude $\Delta V_{1}$ with different fault distances (**c**) The proposed method results with the fault case at 15 km.

This point is extensively studied by checking the criteria in^1^ and^2^ under other conditions. Different cases of forward phase-to-ground fault with fault resistance of 0.1 Ω have been conducted with changing the prefault power factor and fault distance. The change of the positive-sequence current’s phase angle $\Delta \emptyset$, and the change in the magnitude of the positive-sequence voltage $\Delta \left| {V_{1} } \right|$ are presented in Fig. 18a, b, respectively. As shown in Fig. 18a, the prefault power factor condition affects the value of $\Delta \emptyset$ for the same fault distance. As an example, with the close fault conditions at 15 km from the relay point, if the prefault power factor is lagging with a value lower than almost 0.96, $\Delta \emptyset$ turns to be positive instead of being negative. With the tested forward fault cases, a positive $\Delta \emptyset$ combined with a negative $\Delta \left| {V_{1} } \right|$ leads to a misleading decision, indicating a false reverse fault diagnosis. The change of the magnitude of the positive-sequence voltage $\Delta \left| {V_{1} } \right|$ is presented in Fig. 18b where it is negative under all considered test cases. As another example, if the fault is forward at 45 km away from the relay and the prefault power factor is lagging with a value lower than 0.87, the fault direction would be incorrectly identified as well.

This is a significant limitation point in the presented methods in^1^ and^2^. In comparison, the proposed method in this paper is tested for the same faut case at 15 km with different prefault leading and lagging power factor conditions. As shown in Fig. 18c, the locus of the calculated impedance settles in the third quadrant without being negatively affected and consequently the fault direction is correctly identified as forward.

**Fig. 19:**
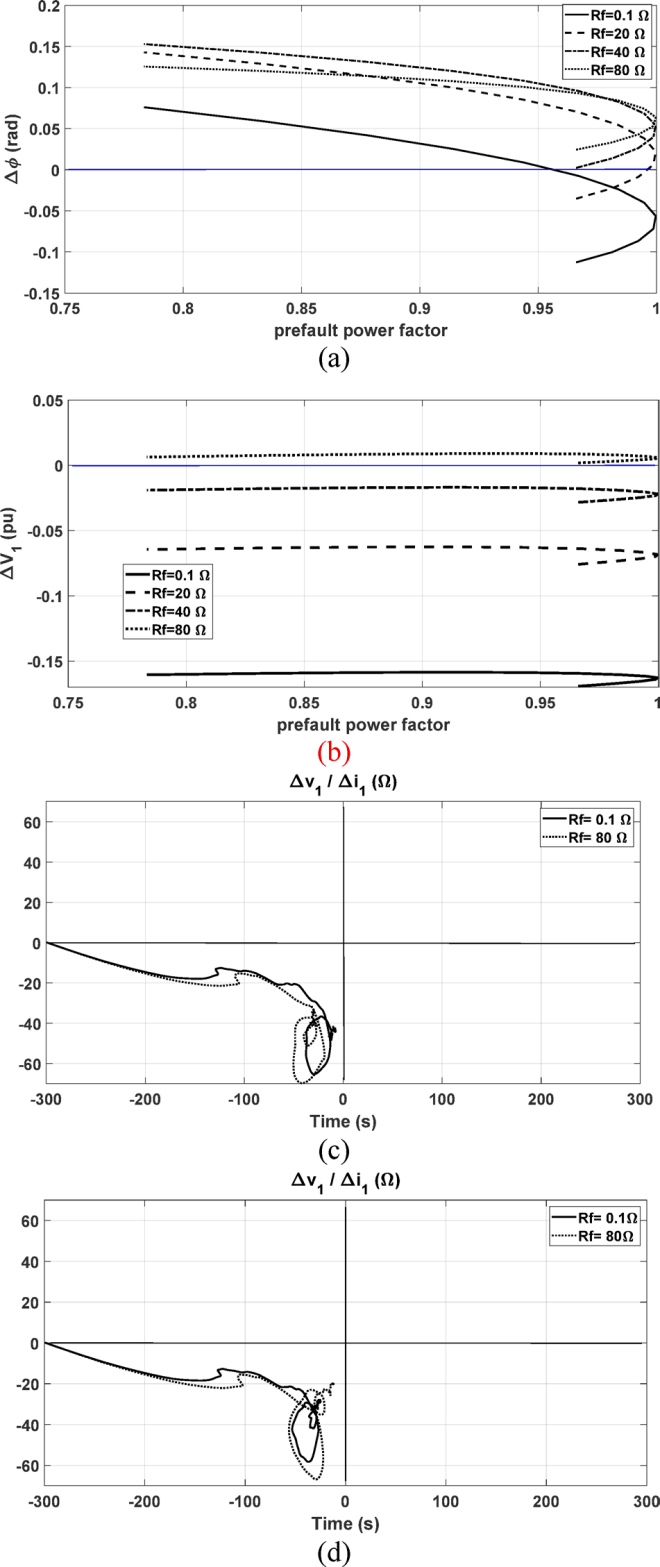
Comparison results considering different fault resistances and under different prefault power factor values with the fault at 15 km; (**a**) The change in the positive-sequence current’s phase angle $\Delta \emptyset$; (**b**) The change of the positive-sequence voltage’s magnitude $\Delta V_{1}$; (**c**) The proposed method results with 0.96 prefault leading power factor at 0.1 and 80 Ω ; (**d**) The proposed method results with 0.78 prefault lagging power factor at 0.1 and 80 Ω .

Further, the performance is checked under different fault resistances. A forward line-to-ground fault at 15 km from the relay point is simulated with different fault resistances. The change the positive-sequence current’s phase angle $\Delta \varnothing ,$ and the change in the magnitude of the positive-sequence voltage $\Delta \left|{V}_{1}\right|$ are presented in Fig. 19a, b, respectively. As shown, $\Delta \varnothing$ tends to be positive value if the fault resistance is increased and the negative change of $\Delta \left|{V}_{1}\right|$ is reduced with increasing the fault resistance value. The change of $\Delta \left|{V}_{1}\right|$ is altered to be positive at higher fault resistances such as the case with 80 Ω. According to^1^ and^2^, the fault is identified as forward if $\Delta \varnothing$ is negative or if both $\Delta \varnothing \mathrm{and} \Delta {V}_{1}$ are positive as summarized in Table 1. However, these criteria are not met under the fault case with 40 Ω fault resistance.

As shown in Fig. 19a, b, under this fault case, $\Delta \varnothing$ is positive $\mathrm{and} \Delta \left|{V}_{1}\right|$ is negative. This leads to a misleading decision, indicating a false reverse fault diagnosis. This means that, the fault direction could not be correctly identified by the methods in^1^ and^2^ under all possible cases. On the other hand, the proposed method is tested with low and high values of fault resistances including 0.1 and 80 Ω as shown in Fig. 19c, d under different prefault leading and lagging power factor. It is depicted that the fault direction is successfully identified as forward fault regardless of the fault resistance value and the prefault power factor value where the obtained locus of the calculated impedance settles in the third quadrant under all tested cases.

#### Comparison study considering T-connected multiterminal system

**Fig. 20:**
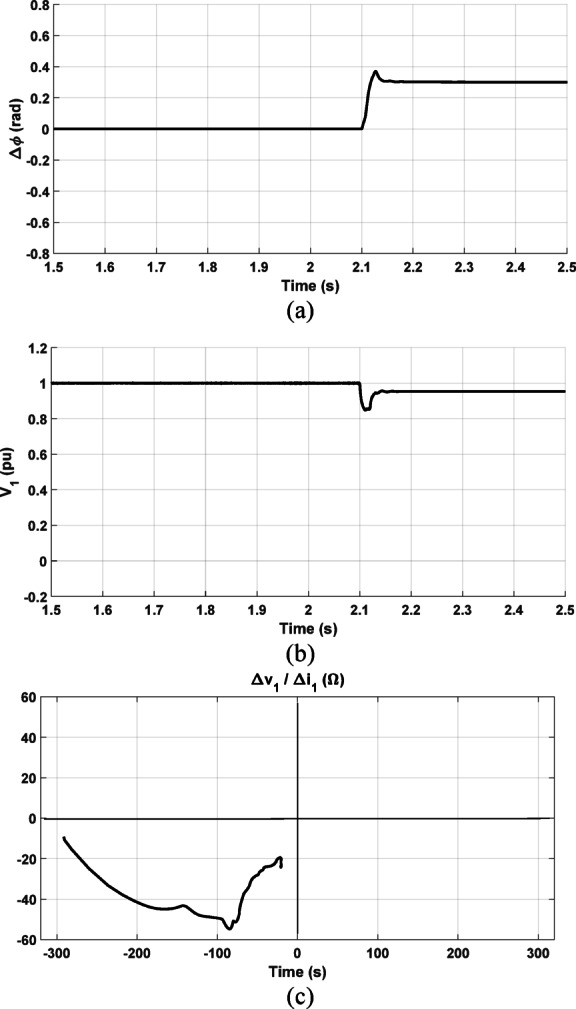
Comparison results considering T-connected multiterminal transmission system (**a**) The change in the positive-sequence current’s phase angle; (**b**) The positive-sequence voltage’s magnitude; (**c**) The proposed method result.

The comparison is continued and validated with different transmission system. The Egyptian T-connected multiterminal system in Fig. 10a is considered. The performance of methods in^1^ and^2^ is tested by checking $\Delta \varnothing$ and $\Delta \left|{V}_{1}\right|$ and compared with the decision of the proposed method in this paper. The prefault power factor is controlled via the integrated wind farms to be lagging where the wind farms are providing active and reactive power to Qena grid. A forward three-phase fault at *F2* with a fault resistance of 15 Ω is simulated. Figure 20a shows the alteration of the positive-sequence current angle $\Delta \varnothing ,$ and Fig. 20b displays the positive-sequence voltage’s magnitude $\Delta \left|{V}_{1}\right| ,$ where the fault occurrence instant is at 2.1 s. As observed in both figures, ∆∅ is positive and $\Delta \left|{V}_{1}\right|$ is negative. According to the criteria presented in^1^ and^2^, the fault direction is indicated as a reverse fault which is a false decision. However, the proposed scheme succeeds in identifying the fault direction as a forward fault. As shown in Fig. 20c, the obtained locus of the positive-sequence impedance is in the third quadrant which means that the fault is forward.

#### Reversed prefault active power flow condition

**Fig. 21:**
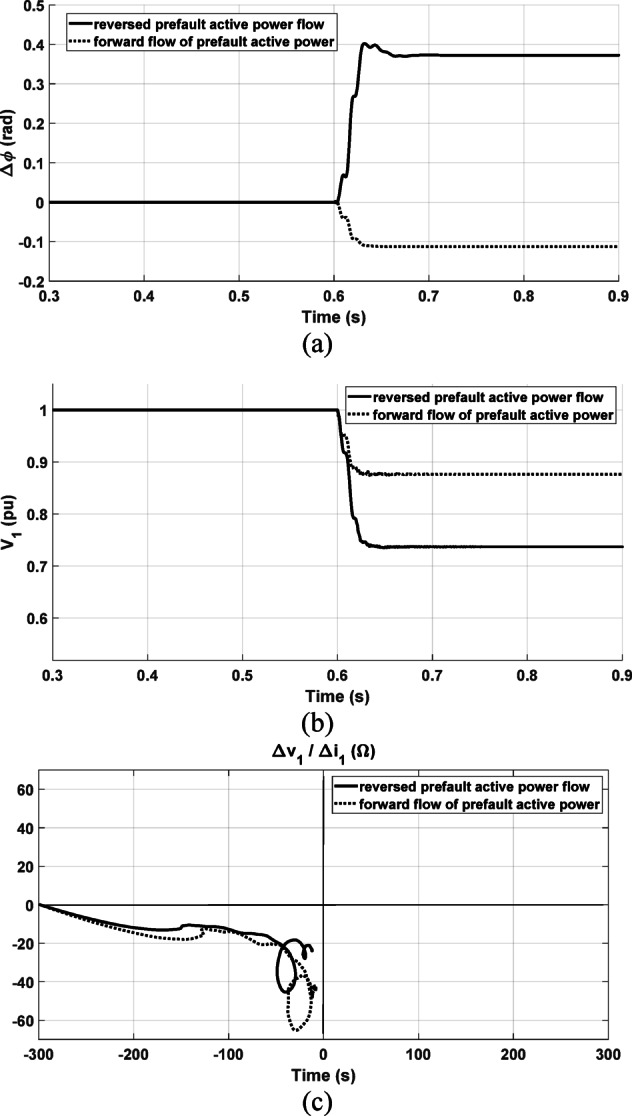
Response with reversed prefault active power flow; (**a**) The change in the positive-sequence current’s phase angle; (**b**) The positive-sequence voltage’s magnitude; (**c**) The proposed method result.

The presented schemes in^1^ and^2^ are affected by the prefault active power flow direction where all conditions need to be altered. In this section, the response of both the proposed scheme and the presented in^1^ and^2^ are investigated under reversed prefault active power flow condition. A forward solid phase-to-ground fault is simulated at 15 km from the relay point in the test system in Fig. 7a. The variables $\Delta \varnothing$ and $\Delta {V}_{1}$ are checked with changing the prefault active power flow direction with the same fault case. As shown in Fig. 21a, b, $\Delta \emptyset$ is altered to positive instead of being negative while $\Delta \left|{V}_{1}\right|$ is still negative. This requires changing the criteria based on the direction of the prefault active power which affects the reliability of the directional relay. However, the proposed scheme has the same response as depicted in Fig. 21c. As seen, it is not affected by the power flow direction. This is a great advantage of the proposed method.

## Reliability evaluation

For evaluating the reliability of the directional relays, many test cases are simulated under different conditions. The reliability evaluation includes assessing the dependability and security. The dependability of the protective relays is the extent of assurance that the relay will operate accurately under forward faults. It is the percentage of accurate trips provided by the relay compared to the desired trips. The dependability is evaluated by the following^34^:

15 $$Dependability\% = \frac{number of correct trips}{{number of desired trips}}*100$$

Further, the security of the directional relaying algorithm is evaluated. This parameter represents the level of assurance that the relay will not give incorrect trip decision with reverse faults. The security is calculated by the following equation^34^:

16 $$Security\% = \frac{number of correct trips}{{number of total trips}}*100$$

Reliability can be understood as the capability of the relay to perform its function without experiencing failed decisions. It is calculated as follows^34^:

17 $$Reliability\% = \frac{number of correct trips*100}{{no. of desired trips + no. of incorrect trips}}$$

240 test cases are simulated under different fault scenarios. All fault types are tested with changing the fault location including 15, 45, 75, 105, and 135 km. In addition, the conducted tests are performed with different values of the fault resistance including 0.1, 20, 40, 60, 80, 100, 120, and 140 Ω. The reliability of both the proposed scheme and the presented methods in^1^ and^2^ are evaluated according to the obtained results from the conducted test cases.

Table 2 shows the obtained results when evaluating the presented methods in^1^ and^2^ under different forward fault cases. The 240 fault cases are tested with changing the prefult power factor. The presented methods in^1^ and^2^ gives the highest reliability under prefault power factor of 0.966 leading with the transmission system 50% compensated. As shown, for each fault type, 40 test cases are conducted. Under three-phase fault cases, the methods in^1^ and^2^ gives 34 correct trips while 6 cases are not identified as forward faults. The same number of correct trips is obtained under phase-to-ground fault conditions but with different fault conditions; and under phase-to-phase faults, 33 cases are correctly identified as forward faults while 7 cases are diagnosed incorrectly as reverse faults. Based on the obtained results of the simulated 120 forward fault cases, the dependability of the presented methods in^1^ and^2^ is 84.16% as declared in Table 3.

**Table 2:** Cases of forward faults are not detected by^1^ and^2^.

	Fault location					
		15 km	45 km	75 km	105 km	135 km
*0.966 leading power factor*						
*50% Compensation*						
*Forward three-phase fault*						
Fault resistance	0.1 Ω					
	20 Ω					
	40 Ω					
	60 Ω		x			
	80 Ω		x			
	100 Ω		x	x		
	120 Ω			x		
	140 Ω			x		

	Fault location					
		15 km	45 km	75 km	105 km	135 km
*Forward phase-to-ground fault*						
Fault resistance	0.1 Ω					
	20 Ω					
	40 Ω	x				
	60 Ω	x				
	80 Ω		x			
	100 Ω		x			
	120 Ω		x			
	140 Ω		x			

	Fault location					
		15 km	45 km	75 km	105 km	135 km
*Forward phase-to-phase fault*						
Fault resistance	0.1 Ω					
	20 Ω					
	40 Ω					
	60 Ω		x			
	80 Ω		x			
	100 Ω		x	x		
	120 Ω		x	x		
	140 Ω			x

**Table 3:** Reliability evaluation of the methods in^1,2^ compared to the proposed method.

	Methods in^1^ and^2^ (%)	Proposed method (%)
$\mathrm{Dependability\%}$	84.1	100
$\mathrm{Security\%}$	100	100
$\mathrm{Reliability\%}$	84.16	100

For evaluating the security, 120 reverse fault cases are tested. The presented methods in^1^ and^2^ do not give any incorrect trip decision. Thus, the security is 100% for the presented methods in^1^ and^2^ as declared in Table 3.

**Fig. 22:**
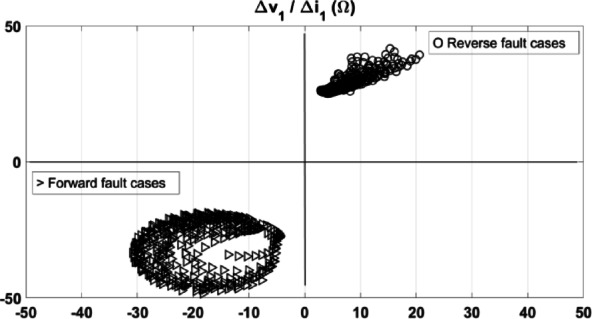
The proposed method results for evaluating the reliability for the tested fault cases.

The reliability of the proposed method is evaluated for the same tested fault cases. The loci of the calculated impedance are presented in Fig. 22 at 2 cycles after fault inception. As shown, the locus of the calculated impedance under the tested forward and reverse fault cases is settled in the third quadrant, and first quadrant, respectively. The obtained results do not include any failed decision. Thus, the reliability of the proposed method is 100% compared to the methods in^1^ and^2^ as declared in Table 3.

## Conclusion

A comprehensive directional relaying algorithm has been proposed for series-compensated transmission systems. The obtained results have demonstrated the robust performance of the proposed algorithm in accurately identifying fault direction under various conditions. Notably, the fault type does not negatively affect the algorithm’s performance due to its dependence on the change in the positive-sequence circuit. Extensive testing with different fault resistance values, up to 3 kΩ, has confirmed the algorithm’s reliability. The proposed scheme successfully identifies fault direction under different fault locations in both two-terminal and T-connected multiterminal transmission systems. Additionally, varying compensation ratio over wide range has been tested, and the results consistently support the accuracy of the relay’s decision. Comparing the proposed scheme to relevant methods in the literature, it stands out for its more reliable performance, particularly considering different prefault power factor values and prefault active power flow direction. Overall, the proposed directional relaying algorithm offers an effective and dependable solution for fault direction determination in series-compensated transmission systems.

## Supplementary Information

Below is the link to the electronic supplementary material.

Supplementary Material 1

## Data Availability

All data generated or analyzed during this study are included in this published article.

## Abbreviations

$\Delta {V}_{1}$: Phasor value of the change of the positive-sequence voltage at the relay
$${\delta }_{1}$$: Angle of $$\Delta {V}_{1}$$
$\Delta \left| {V_{1} } \right|$: The change of the magnitude of the positive-sequence voltage at the relay
$\Delta I_{1}$: Phasor value of the change of the positive-sequence current at the relay
$$\left| {\Delta I_{1} } \right|$$: Magnitude of $$\Delta {I}_{1}$$
$${\theta }_{1}$$: Angle of $$\Delta {I}_{1}$$
${V}_{pre}$: Prefault voltage at fault point
$${I}_{pre}$$: Prefault current
$${\varnothing }_{1}$$: Angle of $${I}_{pre}$$
${I}_{1}$: Postfault positive-sequence current measured by the relay
$${\varnothing }_{2}$$: Angle of $${I}_{1}$$
$\Delta \varnothing$: Change of the angle of the positive-sequence current measured by the relay
${X}_{{L}_{upst.}}$: Equivalent inductive reactance upstream the relay under forward fault
${X}_{{L}_{fault.}}$: Equivalent inductive reactance downstream the relay up to the fault point under forward fault
TL: Transmission line
SCTL: Series-compensated transmission line
SC: Series capacitor
MOV: Metal oxide varistor
RF: Fault resistance
RG: Ground fault resistance
RSC: Rotor side converter
GSC: Grid side converter
d,q: Direct and quadrature components of the signals
P: Output active power from DFIG
Q: Output reactive power from DFIG
QGSC: Output reactive power from the grid side converter
Vdc: DC link voltage in DFIG system
Te: Electromechanical torque
RrLr: Machine rotor resistance and inductance
RsLs: Machine stator resistance and inductance
is: Stator current
ir: Rotor current
PWM: Pulse width modulation
PI: Proportional and Integral controller
