# Supplementary material for: Reliable directional relaying for T-Connected Series-Compensated transmission lines
Source: Sci Rep. 2026 Jan 17;16:2616. doi: 10.1038/s41598-025-33564-9 (PMC12819512; doi:10.1038/s41598-025-33564-9)
Supplement: Supplementary file 1 — Supplementary Material 1 [file 41598_2025_33564_MOESM1_ESM.docx]

# Appendices

*A. Simulated Grid Parameters*

The details of the parameters of the simulated two-terminal system are as follows. The parameters of the simulated grid are depicted in Table S1.

TABLE S1

The parameters of the simulated grid sources

| voltage level (kV) | | 400 |
| --- | --- | --- |
| Frequency (Hz) | | 50 |
| Positive-sequence parameters | resistance (Ω) | 0.0656 |
|  | inductance (mH) | 6.3873 |
| Zero-sequence parameters | resistance (Ω) | 0.2116 |
|  | inductance (mH) | 19.809 |

*B. Simulated Transmission Circuit Parameters*

The parameters of the transmission system are depicted in Table S2.

TABLE S2

The parameters of the simulated transmission circuit

| Positive-sequence parameters | resistance (Ω/km) | 0.0367 |
| --- | --- | --- |
|  | inductance (mH/km) | 1.0139 |
|  | capacitance (µF/km) | 0.01136 |
| Zero-sequence parameters | resistance (Ω/km) | 0.257 |
|  | inductance (mH/km) | 3.73745 |
|  | capacitance (µF/km) | 0.00768 |

*C. MOV Model Parameters*

The MOV is modelled as a controlled current source in parallel with the capacitor as illustrated in Fig. S1. The MOV’s current is determined as a function of the voltage of the series capacitor and the reference voltage [2]. The reference voltage value is 150 kV, p=1000, and q=23.

$$i_{MOV}=P\left( \frac{v}{v_{ref}} \right)^{q}$$


Fig. S1. MOV model.

*D. Details of the simulated DFIG power plant*

The simulated wind power plant is based on a Doubly Fed Induction Generator (DFIG) configuration. The ‎overall structure of the DFIG-based wind power plant is illustrated in Fig. S2(a), which includes ‎both the Rotor Side Converter (RSC) and the Grid Side Converter (GSC). The control schemes for both ‎converters are also depicted, demonstrating how the RSC regulates rotor currents to control active and ‎reactive power, while the GSC maintains DC-link voltage and ensures grid-side power quality.‎ As shown in Fig. S2(b) and S2(c), the standard control scheme of the DFIG is employed. The active power output of the wind plant is regulated based on wind conditions using a maximum power point tracking (MPPT) algorithm. Meanwhile, the reactive power output is controlled to comply with grid code requirements, ensuring proper voltage support and system stability. Proportional and Integral (PI) controllers are utilized for both RSC and GSC control. The gains of PI controllers for the RSC and GSC are listed in Table S3.

TABLE S3

The gains of PI controllers in DFIG system

| Output power PI controller | kp | 3 |
| --- | --- | --- |
|  | ki | 0.6 |
| Rotor current PI controller | kp | 0.59 |
|  | ki | 8 |
|  | kp | 7.9 |
| DC voltage PI controller | ki | 400 |
|  | kp | 0.05 |
| GSC reactive power PI controller | ki | 20 |
|  | kp | 0.83 |
| GSC current PI controller | ki | 5 |

(a)

(b)

(c)

Fig. S2. Doubly Fed Induction Generator (DFIG)-based wind power plant; (a) the configuration of DFIG; (b) The rotor side converter control; (c) the grid side converter control.
